# Structural basis for tRNA-dependent cysteine biosynthesis

**DOI:** 10.1038/s41467-017-01543-y

**Published:** 2017-11-15

**Authors:** Meirong Chen, Koji Kato, Yume Kubo, Yoshikazu Tanaka, Yuchen Liu, Feng Long, William B. Whitman, Pascal Lill, Christos Gatsogiannis, Stefan Raunser, Nobutaka Shimizu, Akira Shinoda, Akiyoshi Nakamura, Isao Tanaka, Min Yao

**Affiliations:** 1 Graduate School of Life Science, Hokkaido University, Sapporo, 060-0810 Japan; 2 Faculty of Advanced Life Science, Hokkaido University, Sapporo, 060-0810 Japan; 3 Japan Science and Technology Agency, PRESTO, Sapporo, 060-0810 Japan; 4 Department of Biological Sciences, Louisiana State University, Baton Rouge, LA 70803 USA; 5 Department of Microbiology, University of Georgia, Athens, GA 30602 USA; 6 Department of Structural Biochemistry, Max Planck Institute of Molecular Physiology, Dortmund, 44227 Germany; 7 Photon Factory, Institute of Materials Structure Science, High Energy Accelerator Research Organization (KEK), Tsukuba, 305-0801 Japan; 8 Bioproduction Research Institute, National Institute of Advanced Industrial Science and Technology (AIST), Sapporo, 062-8517 Japan

## Abstract

Cysteine can be synthesized by tRNA-dependent mechanism using a two-step indirect pathway, where *O*-phosphoseryl-tRNA synthetase (SepRS) catalyzes the ligation of a mismatching *O*-phosphoserine (Sep) to tRNA^Cys^ followed by the conversion of tRNA-bounded Sep into cysteine by Sep-tRNA:Cys-tRNA synthase (SepCysS). In ancestral methanogens, a third protein SepCysE forms a bridge between the two enzymes to create a ternary complex named the transsulfursome. By combination of X-ray crystallography, SAXS and EM, together with biochemical evidences, here we show that the three domains of SepCysE each bind SepRS, SepCysS, and tRNA^Cys^, respectively, which mediates the dynamic architecture of the transsulfursome and thus enables a global long-range channeling of tRNA^Cys^ between SepRS and SepCysS distant active sites. This channeling mechanism could facilitate the consecutive reactions of the two-step indirect pathway of Cys-tRNA^Cys^ synthesis (tRNA-dependent cysteine biosynthesis) to prevent challenge of translational fidelity, and may reflect the mechanism that cysteine was originally added into genetic code.

## Abstract (web summary)

tRNA-dependent cysteine biosynthesis is catalyzed by the transsulfursome protein complex. Here, the authors use a multidisciplinary approach to structurally characterize the archaeal transsulfursome and propose a model for tRNA channeling in the complex.

## Introduction

The fidelity of protein synthesis is based on the accurate aminoacylation of tRNAs in which specific amino acids are attached to cognate tRNAs. A group of enzymes named aminoacyl-tRNA synthetases (aaRSs) are responsible for this reaction. It was believed that there is a matching aaRS for each of the 20 amino acids, which specifically ligates an amino acid to the 3′ terminal adenosine of its cognate tRNA^1, 2^. However, the advent of whole genome sequencing has revealed that several assigned aaRSs are absent in some organisms^3, 4^. In these cases, a non-cognate amino acid is initially attached to the tRNA by the first enzyme, which is subsequently converted to the cognate amino acid by the second enzyme in a tRNA-dependent manner. This indirect pathway is adopted for the synthesis of Gln-tRNA^Gln^ and Asn-tRNA^Asn^ in many bacteria and archaea^5–7^, Cys-tRNA^Cys^ in most of the methanogenic archaea^8^ and Sec-tRNA^Sec^ in many species across all three domains of life^9^. Interestingly, the presence of the indirect pathways is accompanied with the absence of the enzymes responsible for canonical amino acids biosynthesis, where the indirect pathways are the sources of the amino acids biosynthesis by tRNA-dependent mechanism^8^.

The synthesis of Cys-tRNA^Cys^ (and also cysteine) by an indirect pathway involves two enzymes, *O*-phosphoseryl-tRNA synthetase (SepRS) and Sep-tRNA:Cys-tRNA synthase (SepCysS), which are respectively responsible for the attachment of a non-canonical amino acid *O*-phosphoserine (Sep) to tRNA^Cys^ and the conversion of Sep-tRNA^Cys^ to Cys-tRNA^Cys^ ^8^. Phylogenetic studies have suggested that the indirect pathway for Cys-tRNA^Cys^ synthesis is of ancient origin^10^. This system also acts as the sole route for cysteine biosynthesis for these organisms^8^. More recently, it was reported that a third protein SepCysE (SepRS/SepCysS pathway enhancer) is necessary for coupling the consecutive two reactions and for enhancing tRNA^Cys^ binding^11^. Thus, the SepRS, SepCysE, and SepCysS ternary complex (named the transsulfursome) is responsible for the whole process. SepCysE is preserved in class I methanogens, which evolved before other methanogens and may have appeared as the ancestral Euryarchaeota^11, 12^. Therefore, elucidation of the functional mechanism of this system is of vital importance as it may explain the mechanism by which cysteine was added to the genetic code.

SepRS is a homotetramer and structurally characterized as a Class II aaRS, closely related to PheRS^10, 13, 14^. SepCysS is a homodimer with pyridoxal-5′-phosphate (PLP) bound in the active sites that shares structural similarity to the PLP-dependent cysteine desulfurase^10, 15^. The structural analysis of SepCysS in complex with the N-terminal domain of SepCysE (SepCysE(NTD)) showed that the dimer of SepCysE is bound to the dimer of SepCysS with their dimer axes aligned^11^. However, it is not yet known how SepRS is bound to this binary complex to form a ternary complex (transsulfursome) and how Sep-tRNA^Cys^ is transferred between these two enzymes safely. Furthermore, while structural and biochemical analyses showed the recognition of tRNA^Cys^ by SepRS^13, 16, 17^, the specific interaction between SepCysS and tRNA^Cys^ is unclear, which is essential to guarantee the fidelity of the two-step indirect pathway in translation.

In the present study, the structures of the binary complex of SepRS and SepCysE, and the ternary complex of SepCysE, SepCysS, and tRNA^Cys^ from *Methanocaldococcus jannaschii* have been solved. Combined with the SAXS, EM, and biochemical results, the recognition of tRNA^Cys^ by SepCysS was determined and the architecture of the transsulfursome was established. On the basis of these results, we propose a mechanism for tRNA channeling, whereby a hinge motion of the SepCysE-SepCysS complex relative to SepRS and a swinging motion of SepCysE(CTD) with bound tRNA^Cys^ facilitate the transport of the mischarged Sep-tRNA^Cys^ to the second, distant active site.

## Results

### Structure of the SepCysE-SepCysS-tRNA^Cys^ ternary complex

**Fig. 1:**
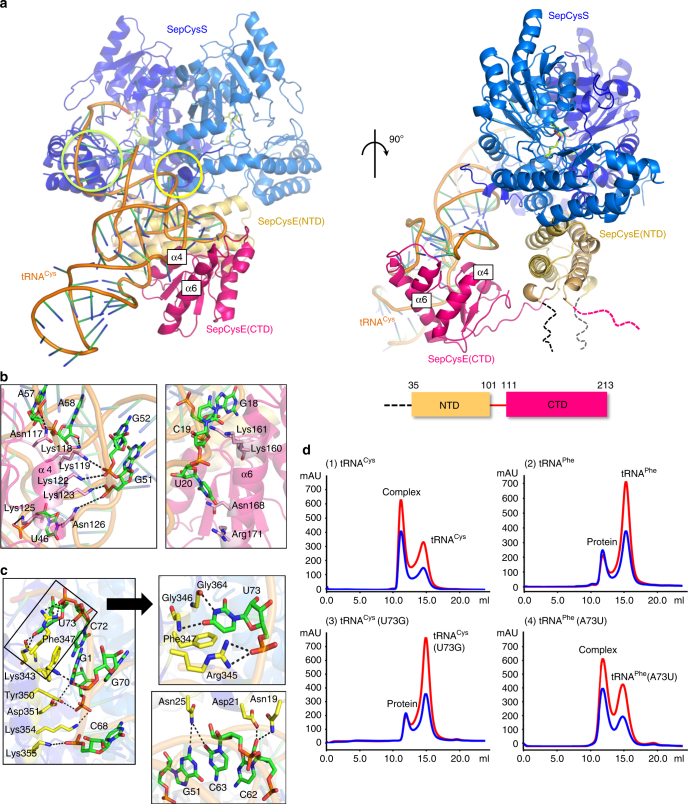
Structure of the SepCysE-SepCysS-tRNA^Cys^ ternary complex. **a** Orthogonal views of the ternary complex drawn in ribbon model. A dimer of the SepCysE N-terminal domain (SepCysE(NTD), yellow orange) is tightly bound to a dimer of SepCysS (blue) with their two-fold axes aligned. From one of the NTDs of the SepCysE dimer, a linker chain extends to the C-terminal domain of SepCysE (SepCysE(CTD), red), which in turn binds to the elbow region of tRNA^Cys^ (orange). The CCA terminus of tRNA^Cys^ is in contact with the active site of SepCysS. A stick representation of PLP is also drawn at the active site. Black and red dashed lines represent flexible loops connected to the disordered N-helix and CTD of SepCysE, respectively. A schematic representation of SepCysE with domain boundaries is given at the bottom. **b** Close-up views of interactions between SepCysE(CTD) and tRNA^Cys^. Interactions are mainly nonspecific between main-chain phosphates of tRNA^Cys^ and lysyl side-chains at α4 and α6 of SepCysE(CTD). Hydrogen bonds are indicated by dashed lines. **c** A detailed view of the recognition of tRNA^Cys^ by SepCysS. The tRNA^Cys^ is bound to SepCysS at two regions marked by yellow/green circles in **a**. Left panel shows the interactions at the helix-loop-helix-loop region (green circle in **a**). Middle panel shows a close-up of the U73 recognition site. Right panel shows the interactions at the N-terminal chain (yellow circle in **a**). **d** Binding assays of the SepCysE-SepCysS complex to tRNA variants by gel filtration. The result shows the importance of the discriminator base U73. Blue and red lines represent the absorption at a wavelength of 280 nm and of 260 nm, respectively

A crystal structure of the SepCysE-SepCysS-tRNA^Cys^ ternary complex was determined at a resolution of 2.6 Å by a molecular replacement method (Table 1, Supplementary Fig. 1a). A Fo-Fc map calculated after several rounds of refinement cycle showed a bulk of density adjacent to the end of one SepCysE(NTD), which represents the linker chain and the C-terminal domain of SepCysE [SepCysE(CTD)], which was disordered in the binary complex previously determined^11^. Thus, this crystal revealed most of the structural features of SepCysE (Supplementary Fig. 1b), as well as the binding manner with tRNA^Cys^ and SepCysS (Fig. 1a). A dimer of the N-terminal domain of SepCysE [SepCysE(NTD), residues 35–101] was tightly bound to a dimer of SepCysS, with their twofold axes aligned. From one of the NTDs of the SepCysE dimer, a linker chain (residues 102–110) extended to the C-terminal domain of SepCysE [SepCysE(CTD), residues 111–213] to which tRNA^Cys^ was bound with its CCA terminus in contact with SepCysS (Fig. 1a). The SepCysE(CTD) of the second subunit was not visible in the crystal, probably because of the absence of bound tRNA^Cys^. The stoichiometry of SepCysE:SepCysS:tRNA^Cys^ = 2:2:1 is in agreement with the result of electrophoresis mobility shift assay (EMSA)^18^.

**Table 1:** Summary of data collection and refinement statistics

	SepCysE-SepCysS-tRNA^Cys^	Transsulfursome
PDB ID	5X6B	5X6C
*Data collection*		
Beamline	SPring-8 BL41XU	Photon Factory BL-5A
Space group	*P*6_5_22	*I*2_1_3
Unit cell parameters (Å)	*a* = *b* = 107.3, *c* = 551.1	*a* = *b* = *c* = 279.8
Resolution range (Å)	48.2–2.60 (2.69–2.60)	48.0–3.10 (3.20–3.10)
*R*_*meas*_ (%)^a^	10.2 (97.5)	14.0 (79.0)
<I/σ(I)>	18.4 (2.1)	16.2 (2.5)
Completeness (%)	99.7 (98.2)	99.9 (99.5)
Redundancy	11.4 (11.8)	7.6 (7.5)
*Refinement*		
No. of reflections	59,370	65,670
*R*_*work*_ */R*_*free*_ (%)^b^	22.5/26.1	18.5/20.9
No. of atoms		
Macromolecules	9,576	9,182
Ligand/ion	—	72
Water	45	109
B-factors (Å^2^)		
Macromolecules	83.3	44.8
Ligand/ion	—	46.8
Water	58.0	32.1
RMSD from ideal		
Bond lengths (Å)	0.005	0.014
Bond angles (°)	1.09	1.35

Values in parentheses are for the highest resolution shell

^a^ *R*_*meas*_ = Σ_*hkl*_ {*N*(*hkl*)/[*N*(*hkl*)−1]}^1/2^ Σ_*i*_ | *I*_*i*_(*hkl*) – < *I*(*hkl*) > |/Σ_*hkl*_ Σ_*i*_ *I*_*i*_(*hkl*), where < *I*(*hkl*) > and *N*(*hkl*) are the mean intensity of a set of equivalent reflections and the multiplicity, respectively

^b^ *R*_*work*_ = Σ_*hkl*_ ||*F*_*obs*_ *|−|F*_*calc*_ *|| /* Σ_*hkl*_ |*F*_*obs*_|, *R*_*free*_ was calculated for 5% randomly selected test sets that were not used in the refinement

The structure of the SepCysE(CTD) consisted of three-stranded parallel β sheets with four peripheral alpha helices (Supplementary Fig. 1b). The connectivity of the secondary structures was β1, α4, β2, α5, α6, β3, and α7. It is basically an α/β fold with short insertion helix α5. Although the primary sequence of SepCysE(CTD) is not related to any known structures in the PDB, a Dali server search^19^ based on the present structure identified that the fold of SepCysE(CTD) is related to the Toprim (topoisomerase-primase) domain, which is found in proteins related to DNA metabolism such as primases, topoisomerases, nucleases, and DNA repair proteins and believed to be an ancestral domain^20^.

### C-terminal domain of SepCysE non-specifically binds tRNA

SepCysE(CTD) was bound to the elbow region of tRNA^Cys^ mainly via van der Waals and ionic interactions. The shape of the SepCysE(CTD) surface was highly complementary to tRNA^Cys^, and the central region of SepCysE(CTD) consisting of secondary structures of α4, β2, α5, and α6 interacted with the TΨC and D loops of tRNA^Cys^ (Fig. 1a). Especially prominent were the α4 and α6 helices in the groove of the tRNA, and the side-chains in these helices rich in lysine residues extended into this groove (Fig. 1b). Although the poor density map at the region of α6 hindered building of precise model of the side chains, most of the residues involved in binding with tRNA^Cys^ could be assigned. Alanine replacement mutations of these residues [(Asn117, Lys118, and Lys119), (Lys122, Lys123, Lys125, and Asn126) and (Lys159, Lys160 and Lys161)], reduced tRNA-binding ability (Supplementary Fig. 2a). Since most of the interactions of SepCysE(CTD) with tRNA^Cys^ are with the phosphate backbone, SepCysE(CTD) is likely to be a non-specific tRNA binding domain. This idea was confirmed by a binding assay of SepCysE(CTD) with other tRNAs (tRNA^Sec^, tRNA^Phe^ and tRNA^His^), which indicated that SepCysE(CTD) can bind these tRNAs with affinities almost at the same level as tRNA^Cys^ (Supplementary Fig. 3a). Therefore, we characterized SepCysE(CTD) as an ancient, non-specific tRNA binding domain. However, we cannot rule out the possibility that SepCysE(CTD) exhibits binding preference to tRNA^Cys^. It was reported that, in *E*. *coli*, the interaction between ribose-phosphate backbone of tRNA^Cys^ and CysRS is essential for recognition (indirect readout)^21^. The same strategy may also be adopted by the present system.

It was previously noted that SepCysE possessed similar binding affinities as SepRS for tRNA^Cys^, but SepCysS did not show measurable binding to tRNA^Cys^ and the formation of binary complex with SepCysE significantly increased the affinities of SepCysS for tRNA^Cys^ ^11^. In the present ternary complex, tRNA^Cys^ was in contact with both SepCysE(CTD) and SepCysS, while no direct interaction was observed between SepCysE(CTD) and SepCysS (Fig. 1a). Thus, it is possible that a mobile SepCysE(CTD) connected to the SepCysE(NTD)-SepCysS complex by a flexible linker, mediated the binding of tRNA^Cys^ to SepCysS. Interactions of SepCysS to the acceptor stem of tRNA^Cys^ may reinforce the binding of tRNA^Cys^ to the SepCysE-SepCysS complex. Consistent with this model, the tRNA^Cys^ binding affinity of the full complex (SepCysE-SepCysS: K_d = _0.35 µM) is somewhat stronger than that of SepCysE(CTD) (K_d = _0.67 µM), while the affinity of SepCysE(del_CTD)-SepCysS for tRNA^Cys^ could not be detected (Supplementary Fig. 3b).

### SepCysS recognizes the discriminator base U73 of tRNA^Cys^

SepCysS is a pyridoxal-5′-phosphate (PLP)-dependent enzyme and a homodimer with a large domain (about 300 residues, residues Gly43–Lys295) and a small domain (about 100 residues, residues Trp296–Lys396) per monomer. PLP was covalently bound to the side chain of the conserved Lys234 located deep in the cleft at the dimer interface. The inner surface of the cleft was highly positively charged, along which the negatively charged acceptor stem of the tRNA^Cys^ had access to the catalytic site deep in the cleft (Fig. 1c).

Two regions were involved in tRNA^Cys^ binding. First, the N-terminal chain of SepCysS extended over the stem of the TΨC loop of tRNA^Cys^ with three residues (Asn19, Asp21, Asn25) interacting with G51, C62, and C63 (Fig. 1c). Deletion of the 30 N-terminal residues (del_1–30) slightly reduced the binding of tRNA^Cys^ (Supplementary Fig. 2b), suggesting its role in tRNA^Cys^ binding is minor. Nevertheless, it may help direct the acceptor stem of tRNA^Cys^ to the active site. Second, a helix-loop-helix-loop region (Pro333–Glu368) at the tip of the small domain held the acceptor stem of tRNA^Cys^ via hydrogen bonds and electrostatic interactions (Fig. 1c). Although most of the interactions were nonspecific between the basic side chains of SepCysS and the phosphate ions of tRNA^Cys^, the discriminator base U73 was strongly recognized by the residues Arg345, Gly346, Phe347 and Gly364; the side chain of Arg345 interacted with the phosphate moiety of U73, and Gly346 and Gly364 together created room exclusively for U73 by specifically interacting with the uracil, and allowing the phenyl ring of Phe347 to stack with U73 and the G1-C72 base pair (Fig. 1c). These residues together acted as a gate for the discriminator base U73 while excluding non-cognate tRNAs. The residues responsible for the recognition of U73 are conserved in methanogenic archaea species (Supplementary Fig. 4a). Deletion of residues Arg344, Arg345, and Phe347 in the recognition region (del_RRF), and Ala substitution mutations at Gly346, Phe347, and Gly364 of SepCysS, reduced the binding affinity of SepCysE-SepCysS to tRNA^Cys^ (Supplementary Fig. 2b).

An in vivo genetic study of *Methanococcus maripaludis* further confirmed the importance of this region. As *M*. *maripaludis* contains a canonical CysRS^22^ and can take up free cysteine from the medium, the inactivation of the whole indirect pathway by deleting both the *sepS* (encoding SepRS) and the *pscS* (encoding SepCysS) genes was not lethal. As expected, the double deletion ∆*sepRS*∆*sepcysS* mutant strain was a cysteine autotroph (Supplementary Fig. 5a). However, the deletion of *pscS* by itself was lethal^23^, possibly because the accumulation of Sep-tRNA^Cys^ was toxic to the cell. Complementation of the ∆*sepRS*∆*sepcysS* strain with SepRS and wild-type SepCysS expressed from a shuttle vector restored growth without cysteine (Supplementary Fig. 5b). However, attempts to transform the ∆*sepRS*∆*sepcysS* strain with a vector expressing SepRS and SepCysS(del_RRF) did not result in viable colonies (Supplementary Table 1), similarly to the *pscS* deletion experiment. This result suggested that SepCysS(del_RRF) was unable to convert Sep-tRNA^Cys^ to Cys-tRNA^Cys^ and that this mutated region was vital for the recognition of tRNA^Cys^.

A binding assay of SepCysE-SepCysS to tRNA^Cys^ variants confirmed the importance of U73. The tRNA^Cys^(U73G) had a weakened binding affinity to SepCysE-SepCysS, while tRNA^Phe^(A73U) acquired binding ability towards SepCysE-SepCysS (Fig. 1d), suggesting that U73, but not the anticodon of tRNA^Cys^, was the major identity element for SepCysS. U73 in tRNA^Cys^ is conserved in the three domains of life, and has been reported as a discriminator for CysRS and SepRS^16^. Here we found that SepCysS also utilizes this recognition element. The fact that the discriminator base U73 is cross-recognized by SepRS and SepCysS may explain, at least partly, the relative insensitivity of SepRS to mutation of this nucleotide base compared with CysRS^17^.

### SepCysE dimer binds to SepRS by a pair of N-terminal helices

**Fig. 2:**
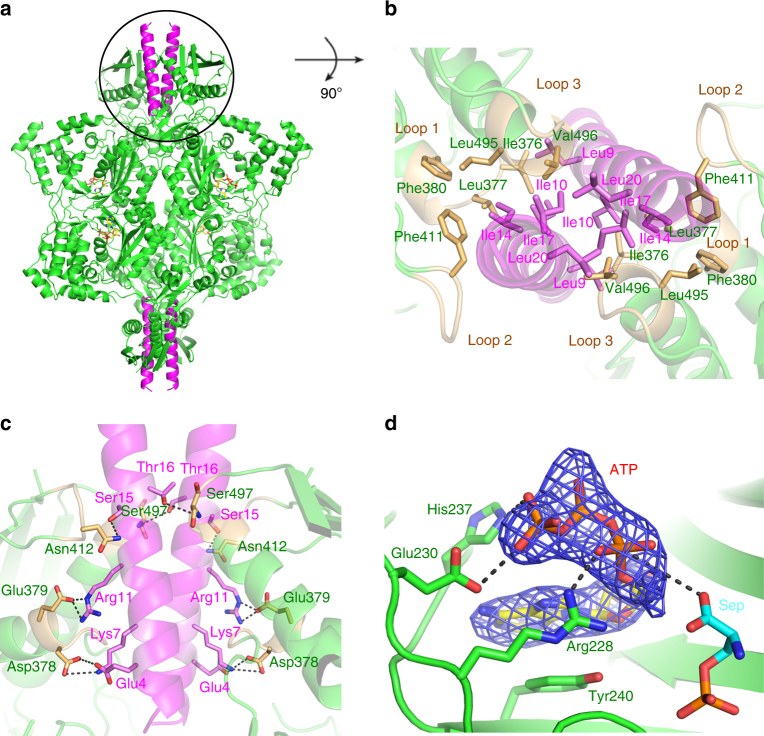
Structure of SepRS in complex with SepCysE. **a** A pair of N-terminal helices (N-helices; (Met1-Lys24)) of the dimeric SepCysE (magenta) wound around each other to form a coiled-coil structure and was tightly bound to the SepRS dimer (green) with their two-fold axes aligned in parallel. ATP in each active site is shown in stick model. **b** Hydrophobic residues of both SepRS and SepCysE contribute to the tight complex formation. **c** Hydrogen bonds also exist between SepRS and the N-terminal helices of SepCysE contributing to the stability of the complex. **d** Bent ATP superposed with *Fo-Fc* omit electron density map contoured at the 2σ level (blue). Arg228, Glu230, His237 and Tyr240 in the active site of SepRS interact with ATP, which adopts a bent conformation conserved in class II aaRSs. Superposition of Sep from SepRS-Sep complex (PDB:2DU3) showed Sep locates next to ATP, with OH group in the reach of α phosphate of ATP to form Sep-AMP

During the study of the quaternary complex SepRS-SepCysE-SepCysS-tRNA^Cys^, we serendipitously obtained the crystal of the ternary complex SepRS-SepCysE-SepCysS. The structure was solved by the molecular replacement method (Table 1). In the electron density map, whole SepRS and the N-terminal helix (Met1–Lys24) of SepCysE(N-helix) were visible (Supplementary Fig. 6a). However, the remaining components of SepCysE and SepCysS were disordered in the crystal, although there was enough space in the unit cell (Supplementary Fig. 6b) to accommodate the entire complex. SepRS was organized as a tetramer with 222 local symmetry (one of the two-fold axes is crystallographic), as previously observed^13, 14^. A pair of N-terminal helices (N-helices) of the dimeric SepCysE wound around each other to form a coiled-coil structure and were tightly bound to the SepRS with their two-fold axes aligned (Fig. 2a). This N-helix was not visible in the structures of the ternary complex SepCysE-SepCysS-tRNA^Cys^, suggesting that an intervening stretch of about 10 residues (Ile25–Ile34) between N-helix and NTD was a flexible linker. This view was consistent with a secondary structure prediction that a long loop connects N-helix and NTD.

The highly conserved hydrophobic residues Leu9, Ile10, Ile17, and Leu20 (Supplementary Fig. 4b) in the N-helix contributed to the hydrophobic interactions within the left-handed coiled-coil structure. Most of these residues (Leu9, Ile10, and Ile17) plus Ile14 were clustered with the hydrophobic residues in three pairs of loops ((loop 1: 376–380 ILDEF), (loop 2: 411–414 FNGE), and (loop 3: 494–498 ALVSN)) locating at the interface of two anticodon domains of the SepRS dimer (Fig. 2b) and contributed to the formation of the complex together with some hydrogen bonds (Fig. 2c). To verify the physiological importance of these interactions, several deletion mutants were made and the binding ability was checked by gel filtration experiments. Whereas deletion mutants of each of the three loops of SepRS showed no significant effect on binding, the double-loop deletion mutants did not bind SepCysE, suggesting these loops together contribute to the interaction with SepCysE (Supplementary Fig. 7). Likewise, N-helix deletion mutant SepCysE(ΔN-helix) completely lost the ability to bind SepRS (Supplementary Fig. 7). This structural and mutational evidence suggested that the SepCysE dimer tightly binds to the SepRS dimer by inserting its coiled-coil N-terminal helices into the interface of the SepRS dimer, which reinforces the stable architecture of transsulfursome.

ATP was observed in the active sites of SepRS. The binding manner of ATP was similar to that of other canonical class II aaRS, especially PheRS^14^. The bent ATP mainly interacted with motif 2 of SepRS via four conserved residues Arg228, Glu230, His237 and Tyr240. Superposition of the structures of SepRS-SepCysE(N-helix) on *Archaeoglobus fulgidus* SepRS-tRNA^Cys^-Sep (PDB entry 2DU3) showed that the OH group of Sep was very close to the α-phosphate of ATP, as necessary to form the phosphoseryl-adenylate (Fig. 2d).

### Dynamic architecture of the transsulfursome

**Fig. 3:**
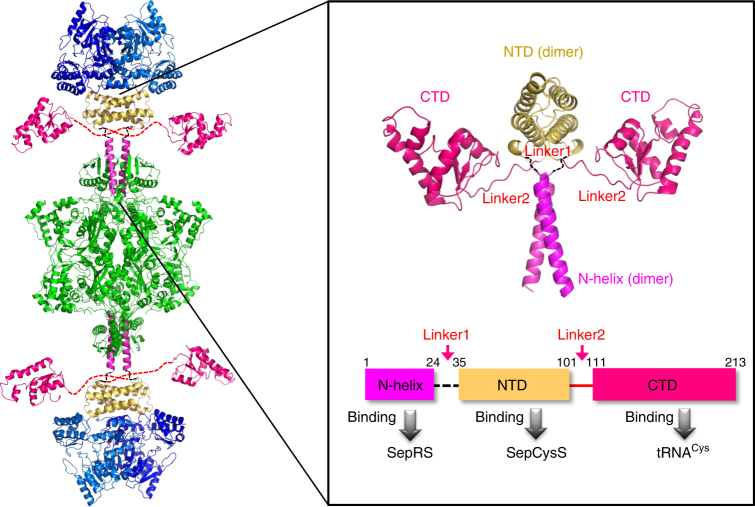
Architecture of transsulfursome. Left, transsulfursome model consisting of tetrameric SepRS (green) at the center, two dimers of SepCysS (blue) at both ends, and SepCysE (magenta, red, yellow) at the position connecting SepRS and SepCysS. Right, SepCysE consists of N-helix (magenta), NTD (yellow), and CTD (red) connected by linker1 (black) and linker2 (red). The three domains of SepCysE (N-helix, NTD, and CTD) each binds SepRS, SepCysS, and tRNA^Cys^, respectively as shown in the schematic representation at the bottom. Note that the orientation of SepCysS relative to SepRS is arbitral due to the flexibility of linker1. The orientation of SepCysE (CTD) is also arbitral due to the flexibility of linker2

Two complex structures solved in the present work were combined to show that SepCysE consisted of an N-terminal helix (N-helix), an N-terminal domain (NTD) and a C-terminal domain (CTD). These components were connected by two linkers, linker1 and linker2, each composed of about 10 residues (Fig. 3, Supplementary Fig. 4b). Each component of SepCysE bound to each of the three molecules, SepRS, SepCysS, and tRNA^Cys^. Both the N-helix and NTD bound as dimers to the dimeric molecules of SepRS or SepCysS, while the CTD worked as “two monomers” of which only one “monomer” bound to monomeric tRNA^Cys^ (Fig. 3). The two intervening linkers (linker1: between N-helix and NTD, and linker2: between NTD and CTD) made SepCysE highly flexible, enabling the movement of the SepCysE(NTD)-SepCysS complex with respect to the SepRS-SepCysE(N-helix) complex and also allowing a large motion of the SepCysE(CTD) with bound tRNA^Cys^ during tRNA transfer from SepRS to SepCysS (see below). The flexibility of SepCysE(NTD)-SepCysS relative to SepRS via linker1 was further confirmed by SEC-SAXS and EM analyses.

**Fig. 4:**
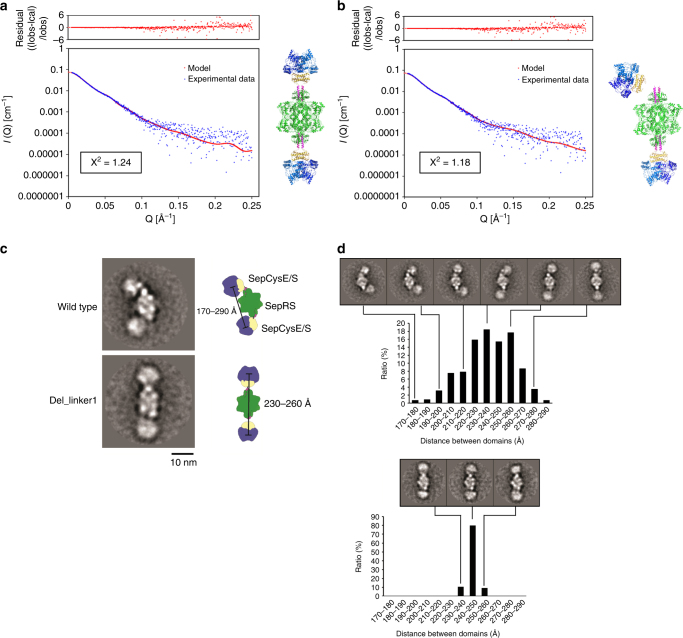
SAXS and EM analyses showing the domain flexibility of the transsulfursome. **a**, **b** SAXS analysis of the transsulfursome. Experimental scattering data (blue) fit well to a theoretical curve (red) calculated from the combined model from crystal structures. Fitting is not sensitive to the azimuth or tilt angles of the (SepCysE(NTD)-SepCysS) domains. **a** Best fitted “straight model” with center-to-center distance of end domains (SepCysE(NTD)-SepCysS) of about 240 Å. **b** Tilted models fit well as long as (SepCysE(NTD)-SepCysS) is within an appropriate distance from SepRS. Given here is one example of a well-fitted “bent model.” **c**, **d** Negative stain EM analysis of wild and mutant (del_linker1) transsulfursome. **c** Selected class-average of a wild type transsulfursome bent (above) and a typical mutant(del_linker1) straight conformation (bottom) (scale bars, 10 nm), with interpretative cartoons showing SepRS, SepCysS and SepCysE in green, blue and khaki, respectively. The cartoons were obtained after graphically mapping a low-pass filtered 3D model of each component into the respective class average. Rulers indicate the distribution range of the total distance between the SepCysE-SepCysS domains at opposite ends of the complex. **d** Center-to-Center distance distribution between the SepCysE-SepCysS domains measured for all class-averages of the wild type transsulfursome (above) and the mutant (del_linker1) (bottom) data set, respectively. Representative class averages are shown and marked by connecting lines. Each class average contains 60–140 single particles. Note that the two SepCysE-SepCysS domains tilt independent from each other and the complex can adopt thereby bent and S-shaped conformations. Typical micrographs and larger sets of class averages are shown in Supplementary Fig. 8

A small angle X-ray scattering (SAXS) experiment was performed to study the structure of the transsulfursome in solution. For simplicity, CTD-truncated SepCysE was used for this experiment. A plot of the intensity of the scattering data (I), as a function of scattering angle (Q) was compared with the transsulfursome model derived from the crystal structures. To take account of the structural variability of the transsulfursome due to flexible linker1, a number of models were generated by changing the distance and orientation of these two rigid structures, and the theoretical curves calculated from the models were compared with the observed plot. As shown in Fig. 4a, the observed plot matched well to a theoretical curve with χ^2^ agreement of 1.24 for “a straight model” after adjusting the distance between the two rigid structures, SepRS-SepCysE(N-helix) and SepCysE(NTD)-SepCysS connected by linker1. Whereas the fitting was sensitive to the distance between the two rigid structures, it was rather insensitive to the tilt angles and even better fitted models were obtained when the angle was also varied (Fig. 4b). Thus, an ensemble distribution of the structures with various tilt angles by linker1 was expected in solution.

The structure of the transsulfursome complex was further characterized by negative stain electron microscopy and class averaging. The particles were adsorbed onto the EM grid in side orientation. Over 20,000 particles were selected from the EM micrographs and subjected to image alignment and classification. This analysis confirmed that the complex possessed an overall architecture, roughly resembling a “three sectional staff”. Tetrameric SepRS was positioned in the center of the complex and dimeric SepCysE-SepCysS at the opposite ends (Fig. 4c). The SepCysE-SepCysS domains did not occupy stable positions but instead flexed independently of the SepRS domain. Thus, the complex could adopt straight and/or curved conformations without changing its overall composition (Fig. 4d). The wild-type complex underwent a tilting-motion from a curved to an S-shaped conformation (Supplementary Fig. 8a, Supplementary Movie 1) and the distribution of the center-to-center distance between the SepCysE-SepCysS components at the opposite ends of the complex ranged from 170 to 290 Å (Fig. 4d). This suggests that a hinge exists at the site of linker1 connecting SepRS-SepCysE(N-helix) and SepCysE(NTD)-SepCysS, and allows movement of these domains relative to each other. Because of the flexibility of the complex and preferred orientation on the EM grid, we were not able to determine a low resolution 3D volume.

To support this notion, we truncated 10 residues of linker1 (transsulfursome (del_linker1)) and analyzed the resulting complex by negative stain EM and image classification. As expected, shortening of the flexible connector, resulted in reduction of the overall flexibility of the complex. Most of the class-averages, obtained from ~ 12,000 particles of this complex, appeared straight and the tilting-motion of SepCysE-SepCysS relative to SepRS was greatly reduced (Fig. 4d, Supplementary Fig. 8b, Supplementary Movie 2). The increased rigidity was also reflected in the narrower distribution of the distance between the SepCysE-SepCysS domains (230–260 Å, Fig. 4d). Thus, motion of SepCysE-SepCysS relative to SepRS is linker1 dependent.

## Discussion

**Fig. 5:**
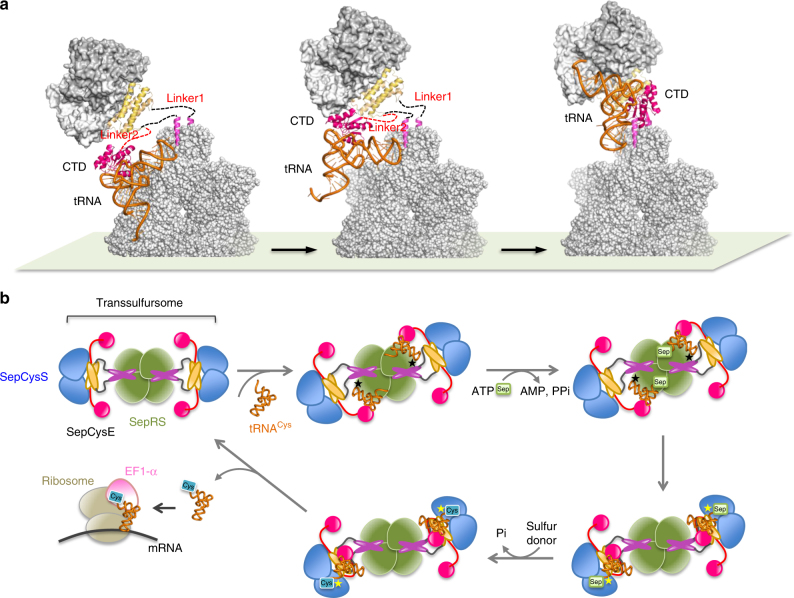
Proposed mechanism of the tRNA-dependent cysteine biosynthesis by transsulfursome. **a** tRNA channeling for consecutive reactions of the transsulfursome. Only upper half of the transsulfursome is drawn here. (Left) The first reaction state (SepRS active form). This model was built by superposing the SepCysE(CTD)-tRNA^Cys^ complex (present work) with the SepRS-tRNA complex (PDB:2DU3). The stretching of linker1 and linker2 allows the SepCysE(CTD)-tRNA^Cys^ complex to reach the binding site on SepRS, while anchoring SepCysE(N-helix) to its binding site on SepRS. The anticodon of tRNA^Cys^ is recognized by the site close to the SepCysE(N-helix) binding site. (Middle) The intermediate state. The mischarged Sep-tRNA^Cys^ moves from the first reaction site on SepRS to the second reaction site on SepCysS without being released from bound SepCysE(CTD). Two flexible linkers of SepCysE allow the movement of Sep-tRNA^Cys^ over this long distance. (Right) The second reaction state (SepCysS active form). The discriminator base of U73 and possibly the Sep moiety of Sep-tRNA^Cys^ are recognized by SepCysS, followed by conversion to the canonical Cys-tRNA^Cys^. The anticodon of tRNA^Cys^ may not be recognized by SepRS in this step. **b** A schematic drawing of the tRNA-dependent cysteine biosynthesis by transsulfursome. N-helix (purple) and NTD (orange) of SepCysE bridge the SepRS (green) and SepCysS (blue) to form ternary complex (transsulfursome) with a molar ratio of 4:4:4. SepCysE(CTD) (red) shuttles tRNA^Cys^ between two distinct active sites on transsulfursome for sequential reactions catalyzed by SepRS and SepCysS. Each of the two enzymes recognizes tRNA^Cys^ separately in the two steps. The released Cys-tRNA^Cys^ is delivered to ribosome by EF-1α to finally incorporate a cysteine into proteins. Two tRNA substrates are bound to the opposite positions on transsulfursome. The recognition sites of tRNA^Cys^ by SepRS and SepCysS are marked with black and yellow asterisks, respectively

**Fig. 6:**
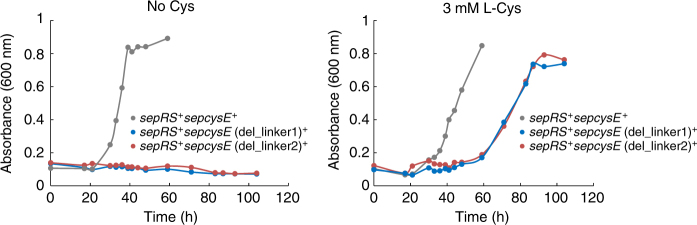
Growth curves of *M*. *maripaludis* variants showing the vital importance of the two linkers of SepCysE. Linker deletion variants are lethal in the absence of Cys (left), which can be rescued by adding 3 mM L-Cys (right) to the medium. This result suggests an essential role of the linkers for the synthesis of Cys-tRNA^Cys^. The inocula were 10^5^ cells per 5 ml culture. Each growth curve is a representative of triplicates. The *sepRS* ^*+*^ *sepcysE* ^*+*^, *sepRS* ^*+*^ *sepcysE*(del_linker1)^*+*^, and *sepRS* ^*+*^ *sepcysE*(del_linker2)^*+*^ strains represent the ∆*sepRS*∆*sepcysE* double deletion mutant strain supplemented with SepRS/SepCysE(WT), SepRS/SepCysE(del_linker1), SepRS/SepCysE(del_linker2) expressed from a vector, respectively. The SepCysE(del_linker1) is a *M*. *maripaludis* SepCysE mutant with Q28-N35 deleted, and the SepCysE(del_linker2) is a SepCysE mutant with T107-Q113 deleted

The ternary complex SepCysE-SepCysS-tRNA^Cys^ solved in the present work presents the structure of which the 3′-end moiety of tRNA^Cys^ is at the active site for the second reaction, while SepRS-tRNA complex solved previously shows the structure for the first reaction^13^. Distance between these two active sites in the transsulfursome is not fixed due to the conformational change caused by the flexible linker1. However, model building shows that the distance is no less than 90 Å. Therefore, tRNA^Cys^ must move a long distance between these two sites during two step reactions. Furthermore, as both SepRS and SepCysS access to the same side of tRNA^Cys^, it is not possible for SepRS and SepCysS to bind to the tRNA^Cys^ simultaneously and to make a ternary complex. In contrast, as SepCysE(CTD) accesses to tRNA^Cys^ from opposite side, it binds to the tRNA^Cys^ without disturbing the binding of the SepRS or SepCysS to tRNA^Cys^. These considerations suggest a scenario for this consecutive reactions where SepCysE(CTD) carries tRNA^Cys^ from the first active site to the second site (Fig. 5a). At first, tRNA^Cys^ bound to SepCysE(CTD) binds to the active site of SepRS while SepCysE(N-helix) remains fixed to SepRS. The model building shows such complex is possible by extending the two linkers of SepCysE (Fig. 5a, left). This is consistent with the experiment that SepCysE enhances the binding of SepRS and tRNA^Cys^ ^11^. After Sep is attached at the first reaction site, CCA terminus of the tRNA^Cys^ leaves from the active site (Fig. 5a, middle) and rotates with the SepCysE(CTD) to reach binding site of SepCysS. The structure of the present ternary complex shows the structure at this stage. SepCysS recognizes the discriminator base U73, and possibly Sep moiety of tRNA^Cys^, and the tRNA^Cys^ separates from the anticodon recognition region of SepRS when CCA terminus binds to the second reaction site (Fig. 5a, right). In this model, two linkers are critically important to satisfy the flexibility required for this long-distance swing of tRNA^Cys^. Therefore, we examined this model by making two SepCysE mutants of which each of two linkers were truncated. The importance of the linkers was studied by an in vivo genetic study. The ∆*sepRS*∆*sepcysE* double deletion mutant strain is a cysteine auxotroph, which can be complemented by expressing wild-type SepRS and SepCysE from a shuttle vector. However, expression of wild-type SepRS together with either SepCysE(del-linker1) or SepCysE(del-linker2) in ∆*sepRS*∆*sepcysE* still required cysteine for growth (Fig. 6). This result indicates that both linkers are essential for the function of the transsulfursome.

The multiple domain architecture of SepCysE confers flexibility to the transsulfursome, which enables SepCysE-SepCysS moiety to bend to SepRS to facilitate the access of tRNA acceptor arm to active site of SepRS. However, this bending keeps SepRS out of reach for another half of the dimeric moiety of SepCysE-SepCysS. That is, the dimeric SepCysE-SepCysS can convey only one tRNA at a time (Fig. 5b). In all, the transsulfursome can afford to catalyze two tRNAs at a time although it possesses four sets of active sites. This is compatible with our result that one transsulfursome only binds two tRNAs (Supplementary Fig. 9) and also to the previous results that the tetrameric SepRS binds two tRNAs and that only two of the four chemically equivalent subunits are active^24^. It is possible that the left and the right active sites of the transsulfursome are alternately used for reaction. As a reference, the alternate mechanism is believed to be adopted by HisRS^25^. The highly interdigitated structure of SepRS may facilitate the alternate catalysis^24^. On the basis of these results, we propose the scheme of the tRNA-dependent cysteine biosynthesis by the indirect pathway (Fig. 5b), where multidomain SepCysE binds SepRS and SepCysS to assemble into a functional unit (transsulfursome) and mediates the transfer of tRNA substrate between the active sites of SepRS and SepCysS via flexible linkers. The released final product Cys-tRNA^Cys^ is delivered to ribosome by EF-1α to incorporate a cysteine into proteins.

Among the indirect pathways known today, the system for Gln/Asn-tRNA^Gln/Asn^ synthesis is best studied. The complex named transamidosome is responsible for this synthesis. The transsulfursome studied here is very different from this system, although both systems are involved in the indirect aminoacylation. First, formation of the transsulfursome is tRNA-independent and could be stable throughout the two reactions, whereas transamidosome does not form a stable complex^26, 27^. This is reasonable because dynamic dissociation of the complex is important for transamidosome, as non-discriminating GluRS/AspRS show dual-specificity to tRNA^Glu^/tRNA^Asp^ and tRNA^Gln^/tRNA^Asn^ ^28, 29^, and the second enzyme amidotransferase (GatCAB) is also employed in the two kinds of transamidosome^30–32^. By contrast, the transsulfursome is specialized for synthesizing Cys-tRNA^Cys^, and stable formation of the transsulfursome is beneficial for the efficient function of this complex in a high-temperature habitat. Second, unlike the case of the transamidosome, both SepRS and SepCysS have access to the same site of the major groove of tRNA^Cys^ to recognize the discriminator base U73^13, 16, 17^. This suggests that the two enzymes evolved independently and only at a later stage they were combined together to form a system for Cys-tRNA^Cys^ synthesis. This scenario implies that it is difficult to form a ternary complex such that the CCA terminus of tRNA is close to both active sites of the two enzymes and that the system requires a large movement of tRNA rather than just flipping the acceptor arm of tRNA between the two active sites, as in the case of transamidosome. Third, whereas in the transamidosome the first enzyme (Glu/AspRS) appears to continuously recognize the anticodon of the cognate tRNA throughout the entire two-step reaction, our model of the transsulfursome suggests that the anticodon is recognized only at the first step. In fact, it is unlikely that SepRS could specifically recognize tRNA^Cys^ in both steps because tRNA^Cys^ needs to be accessed from completely different directions at each step. The recognition of U73 of tRNA^Cys^ by SepCysS also implies that the anticodon recognition by SepRS is redundant in the second step, although we cannot rule out the possibility that tRNA^Cys^ maintains some kind of interaction with SepRS to safeguard the transfer of tRNA^Cys^.

The present structure analysis revealed how SepCysE assembles two enzymes (SepRS and SepCysS) and a substrate (tRNA) together to create a mechanism of aminoacylation of tRNA in the indirect pathway. However, the indirect pathway for aminoacylation of tRNA is associated with the risk that the mischarged intermediate is used for protein synthesis. Therefore, the direct pathway is employed in most extant organisms. Indeed, in some archaeal lineages, CysRS was horizontally transferred and replaced the indirect mechanism^10, 33^. Nonetheless, the ancestral indirect pathway for Cys-tRNA^Cys^ synthesis has been preserved in methanogenic archaea, although the reason for this is unclear. One possible explanation is the energetic advantage by the direct link of amino acid synthesis and protein synthesis in methanogens where cysteine in the proteome is in a large demand^34^. Whatever the reason for this would be, the structure of transsulfursome reflects the most primordial feature of a multiprotein complex each of whose components have acquired individual functions. SepCysE, which is made up of three different binding domains, enhances the efficiency of the two reactions by linking the two enzymes together. By binding the substrate tRNA, it further increases the reaction efficiency while reducing the risk that the mischarged intermediate is released. This complex may not only have facilitated the addition of Cys to the genetic code but also contributed to the evolution of other multi-protein complexes. The architecture of the transsulfursome has the potential to guide protein engineering for site-specific incorporation of the unnatural amino acid *O*-phosphoserine (Sep) into the proteome^35, 36^.

## Methods

### Expression and purification of transsulfursome

The pET-15b vector encoding *M*. *jannaschii* SepRS was transformed into *E*. *coli* strain B834(DE3) plus pRARE2 (Novagen). The pET-15b vector encoding SepCysS together with pCDF_Duet-1-derived plasmid encoding SepCysE were cotransformed into B834(DE3) plus pRARE2. Overexpression of the proteins was induced by 0.5 mM IPTG at 25 °C overnight. For purification of the proteins, cells expressing SepCysE-SepCysS and SepRS were separately sonicated and the extracts were heat-treated at 75 °C for 30 min. The SepCysE-SepCysS was first purified with a HisTrap HP column (GE Healthcare) and eluted with a linear gradient of 20–500 mM imidazole in buffer containing 50 mM HEPES-NaOH (pH 7.5), 300 mM NaCl, and 5 mM MgCl_2_. The collected eluate was then mixed with purified SepRS before loading onto an additional HisTrap HP column (GE Healthcare). The eluted fractions were pooled and purified using a Hitrap Heparin HP column and eluted with a linear gradient of 0.3–1 M of NaCl in the buffer containing 50 mM HEPES-NaOH (pH 7.5) and 5 mM MgCl_2_. The protein fractions were then loaded onto a HiLoad 16/60 Superdex 200 prep grade column (GE Healthcare) equilibrated with buffer containing 20 mM HEPES-NaOH (pH 8.0), 5 mM MgCl_2_, and 10% glycerol. The expression and purification of mutants of the transsulfursome follows the same procedure as described above. The SepCysE-SepCysS complex was purified using the same protocol but without addition of SepRS^18^. Mutagenesis was performed using a QuikChange mutagenesis kit (Agilent Technologies). The information of primers used in mutagenesis are summarized in Supplementary Table 2.

### Preparation of *M*. *jannaschii* tRNA^Cys^

The *M*. *jannaschii* tRNA^Cys^ was transcribed in vitro by T7 polymerase^18^. PCR was performed to synthesize the template DNA for tRNA^Cys^ transcription. The sequence of the DNA oligonucleotides used in PCR were as follows: Forward primer 5′-TAATACGACTCACTATAGCCGGGGTAGTCTAGGGGCTAGGCAGCGGACT-3′; Middle primer 5′-CTAGGCAGCGGACTGCAGATCCGCCTTACGTGGGT-3′; Reverse primer 5′-TGGAGCCGGGGGTGGGATTTGAACCCACGTAAGGC-3′. T7 promoter is underline. After transcription at 37 °C for 5 h, reaction was terminated and product was loaded onto DEAE column. The tRNA^Cys^ was eluted by linear increase of 0.15–2 M NaCl gradients in buffer containing 50 mM MES (pH 6.5), 150 mM NaCl, and 0.2 mM EDTA. The tRNA^Cys^ was further purified by 10% denaturing Urea-PAGE and stored in −30 °C.

### Crystallographic analysis of SepCysE-SepCysS-tRNA^Cys^

SepCysE-SepCysS and tRNA^Cys^ were mixed at a molar ratio of 1:1.2, with a protein concentration of 16 mg ml^−1^. The crystallization drop was set by mixing a 0.75 µL aliquot of the sample with an equivalent volume of reservoir solution. Diffraction quality crystals were obtained in the condition containing 0.1 M MES pH 5.0, and 2.4 M ammonium sulfate^18^. X-ray diffraction of the crystal was collected on a beamline BL41XU at the SPring8 (Harima, Japan), and the data were processed using the XDS and CCP4 packages^18^. The crystal structure of SepCysE-SepCysS-tRNA^Cys^ was solved by the molecular replacement method^18^ and the C-terminal domain of SepCysE was manually constructed with Coot^37^. Initial protein models were fitted manually using Coot and tRNA models were automatically rebuilt by LAFIRE_NAFIT^38^. Structure refinement was performed using phenix.refine^39^. After refinement, R_work_ and R_free_ factors converged to 22.5% and 26.1%, respectively. Statistical information of the structure refinement is summarized in Table 1.

### Crystallization and data collection of transsulfursome

In an attempt to solve the structure of the SepRS-SepCysE-SepCysS-tRNA^Cys^ quaternary complex, 0.75 μL of solution containing 50 μM SepRS-SepCysE-SepCysS(del_RRF), 30 μM tRNA^Cys^, and 10 mM ATP were mixed with an equivalent volume of reservoir (0.1 M Tris–HCl pH 7.0, 0.3 M ammonium sulfate, 36% (w/v) MPD). The well-diffracted crystals were obtained after dehydration trials. Diffraction data were collected on a beamline BL-5A at the Photon Factory (Tsukuba, Japan). The data were processed using the XDS package^40^.

### Structure determination and refinement of transsulfursome

The structure was solved by the molecular replacement method using AutoMR of the Phenix package. The structures of *M*. *jannaschii* SepRS (PDB entry 2DU7), *M*. *jannaschii* SepCysS-SepCysE(NTD) (PDB entry 3WKR), and *E*. *coli* tRNA^Cys^ (PDB entry 1B23) were used as search models. However, only SepRS was found by this search. In the Fo-Fc map subsequently created, a pair of N-terminal helices of SepCysE was obtained. ATP was also found in the active site of SepRS. Structure refinement was performed using phenix.refine with secondary structure restraints, and final R_work_ and R_free_ factors converged to 18.5% and 20.9%, respectively. Statistical information of the structure refinement is summarized in Table 1.

### Isothermal titration calorimetry experiment

Isothermal titration calorimetry (ITC) experiment was performed to investigate the interaction of proteins and tRNA. Protein and tRNA were dialyzed to buffer containing 20 mM HEPES-KOH pH 8.0, 150 mM KCl, 5 mM MgCl_2_, and 10% glycerol. The protein samples were loaded to the cell of Nano ITC (TA Instruments) and 25 aliquots of 2 µL of tRNA^Cys^ were injected into the cell with a time interval of 180 s under a stirring speed of 250 rpm at 60 °C. The data were analyzed using the program NanoAnalyze. The control (buffer to buffer, protein to buffer, and tRNA to buffer) was also performed to normalize the experiment.

### Gel filtration assay

The binding affinity of SepCysE-SepCysS and tRNA mutants was analyzed by gel filtration. The 50 µM protein was incubated with 30 µM tRNA at room temperature for 1 h in the solution containing 20 mM HEPES-KOH pH 8.0, 150 mM KCl, 5 mM MgCl_2_, and 5% glycerol. The sample was loaded onto a Superdex 200 10/300 GL column (GE Healthcare) and the elution profile was monitored at 254 and 280 nm. To investigate the binding between SepRS and SepCysE, 100 µM SepRS variants were incubated with 400 µM SepCysE variants and loaded onto a Superdex 200 10/300 GL column equilibrated with binding buffer containing 20 mM HEPES-NaOH pH 7.5, 300 mM NaCl, and 10% glycerol.

### Electrophoretic mobility shift assay

Electrophoretic mobility shift assay (EMSA) was performed to analyze the binding ratio of proteins and tRNA. For transsulfursome and SepCysE-SepCysS, 10 μM tRNA was mixed with stepwise increases of proteins in the binding buffer containing 20 mM HEPES-KOH, pH 8.0, 150 mM KCl, 5 mM MgCl_2_, and 10% glycerol and incubated at room temperature for 30 min. To charaterize SepCysE(CTD) as a univeral tRNA-binding domain, 10 μM tRNA^Cys^, tRNA^Sec^, tRNA^Phe^ and tRNA^His^ were separately incubated with 80 μM SepCysE(CTD). The samples were loaded onto a 10% non-denaturing polyacrylamide gel and electrophoresis was performed at a constant voltage of 100 V. The gel was separately stained with ethidium bromide and Coomassie brilliant blue.

### Structural analysis in solution by SEC-SAXS

Small angle X-ray scattering incorporated with size exclusion chromatography (SEC-SAXS) was used to analyze the structure of SepCysE(CTD)-truncated transsulfursome in solution. SEC-SAXS data collection was carried out under room temperature at the beamline BL-10C, Photon Factory (Tsukuba, Japan). A wavelength of 1.500 Å was used, and the specimen-to-detector distance was 2,010 mm, as calibrated with silver behenate. Sample was loaded onto a Superdex 200 Increase 10/300 GL column (GE Healthcare) and the collected fractions were directly exposed to X-rays. During data collection, the flow rate was set at 0.05 ml min^−1^ and total 180 X-ray scattering images were collected for 60 min. The scattering images were recorded on a PILATUS3 2 M detector (Dectris) and each two-dimensional scattering image was circularly averaged to convert to the one-dimensional scattering intensity data by using the software SAngler^41^. The pair distribution function P(r) was calculated using DATGNOM4^40^, and all results are summarized in Supplementary Fig. 10 and Supplementary Table 3. Simulation experiment using crystal structure was carried out using CRYSOL programs^42^. The experimental data used for comparison with a theoretical profile is the data measured at the peak position in SEC-SAXS measurement since the interparticle interference was not observed in the serial data of SEC-SAXS.

### Negative stain electron microscopy and image processing

The 4 μL of wild type transsulfursome and mutant (del_linker1) were adsorbed for 45 s at room temperature to freshly glow-discharged carbon-coated copper-grids (Agar scientific; G2400C). After washing with a 10 μL drop of buffer composed of 20 mM HEPES-KOH (pH 8.0), 200 mM KCl, and 5 mM MgCl_2_ and a 10 μL drop of 0.75% freshly prepared uranyl formate solution, the grids were negatively stained with another drop of uranyl formate for 45 s and air-dried. Images were collected using a FEI G Spirit TEM operated at 120 kV, at a nominal magnification of 67k on a 4k × 4k CMOS camera F416 (TVIPS). Particles were selected using e2boxer. The wild type and mutant transsulfursome data sets contained 29,266 and 11,972 single particles, respectively. Because of the overall flexibility of the complex, focused reference-free alignment and classification procedures implemented in SPHIRE^43^ and EMAN2^44^ were applied^45^. In particular, images were first centered and rotationally aligned, using the reference free approach implemented in SPHIRE. Subsequently, a k-means classification was performed, within a mask including only SepRS in the center of the complex and excluding the peripheral and flexible SepCysE-SepCysS densities. In total 10–20 class averages were produced showing sufficient amount of details for SepRS, whereas SepCysE-SepCysS appeared rather blurred. Then we extracted the members of each class-average and performed a second round of k-means classification for each resulting subset, this time using a mask including the complete complex. The final class-averages contained 24–167 members, showed improved features for all components and were used to measure the center-to-center distance between the SepCysE-SepCysS components at the opposite ends of the complex, as shown in Fig. 4c, d. A sequence of selected class-averages in random order was used to create a movie (Supplementary Movie 1 and 2).

### Mutagenesis of *Methanococcusmaripaludis*

The replacement of the *scsS* gene (*mmp1240*, encoding SepCysS) with a puromycin resistance (*pac*) cassette^46^ was generated in the markerless Δ*sepS* strain^11^ by homologous recombination. We confirmed the genotype of the Δ*sepRS/*Δ*sepcysS*::*pac* strain (S700) by Southern hybridization. For expression of SepRS and SepCysS, the two genes, *mmp0688 and mmp1240*, were inserted into the vector pMEV2 under the control of the *hmvA* ^47^ and *sla* promoters, respectively. The *sepRS* ^+^ *sepcysS* ^+^ strain (S702) was then obtained by transformation of S700 with pMEV2-*mmp0688-mmp1240*. The SepCysS(del-RRF) variant was constructed with the plasmid pMEV2-*mmp0688-mmp1240* using the QuikChange mutagenesis kit (Agilent Technologies) and then transformed into strain S700. The mutations of SepCysE (MMP1217), including the variants of SepCysE(del-linker1) and SepCysE(del-linker2), were constructed by using the plasmid pMEV2-*mmp0688-mmp1217* and then transformed into the Δ*sepRS/*Δ*sepcysE*::*pac* strain (S761)^11^.

We grew *M*. *maripaludis* at 37 °C in 28-ml aluminum sealed tubes with 5 ml of McFAA medium (minimal medium + 0.4 M sodium formate + 10 mM sodium acetate + 1 mM L-Ala) buffered with 0.2 M glycylglycine (pH 8.0) and reduced with 3 mM dithiothreitol^11^. L-Cys (3 mM), puromycin (2.5 µg ml^−1^), and neomycin (0.5 mg ml^−1^ in plates and 1 mg ml^−1^ in broth) were added as needed. Antibiotics were omitted when comparing the growth of the WT and mutants. As the sulfur source, 3 mM sodium sulfide was added before inoculation^48^.

### Data availability

Coordinates and structure factors have been deposited in the Protein Data Bank under accession codes PDB 5X6B (SepCysE-SepCysS-tRNA^Cys^ complex) and PDB 5X6C (SepRS-SepCysE complex). Other data are available from the corresponding author upon reasonable request.

## Electronic supplementary material

Supplementary Information

Description of Additional Supplementary Files

Supplementary Movie 1

Supplementary Movie 2
