# Supplementary material for: Structural basis for tRNA-dependent cysteine biosynthesis
Source: Nat Commun. 2017 Nov 15;8:1521. doi: 10.1038/s41467-017-01543-y (PMC5688128; doi:10.1038/s41467-017-01543-y)
Supplement: Supplementary file 1 — Supplementary Information [file 41467_2017_1543_MOESM1_ESM.pdf]

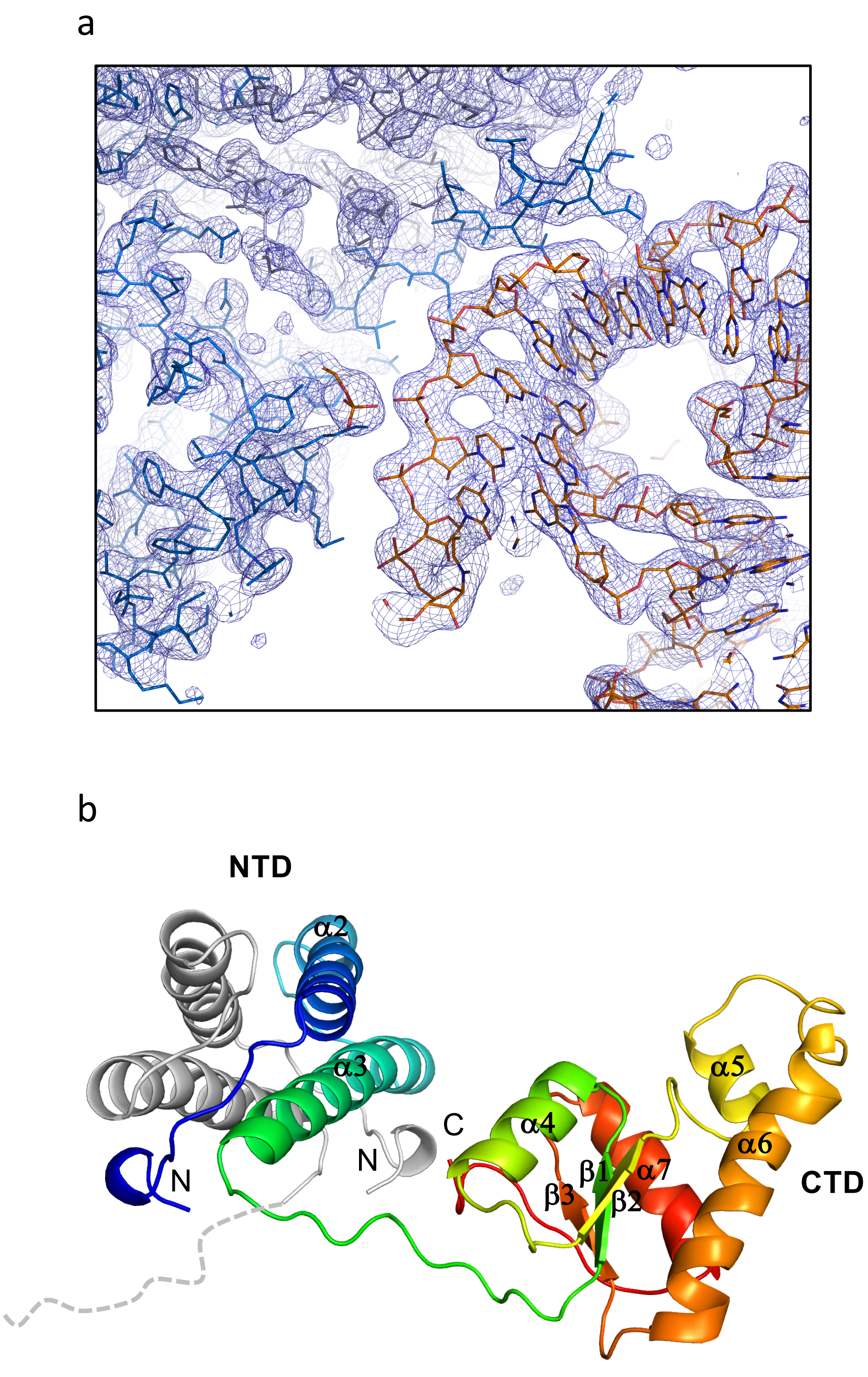

Supplementary Figure 1 Structure of SepCysE-SepCysS-tRNA^{Cys}. (a) Part of the structure of SepCysE-SepCysS-tRNA^{Cys} with 2Fo-Fc density map contoured by 1.0σ. SepCysE(CTD) and tRNA^{Cys} are colored by blue and orange, respectively. (b) Ribbon diagram of the structure of SepCysE in the crystal of the ternary complex. The N-terminal domain (NTD: residues 35 to 101) exists as a dimer from which a polypeptide chain (linker2) extends to a C-terminal domain (CTD: residues 111–213). One protomer is shown with rainbow coloring with secondary structural elements. The other protomer is shown in gray, of which the C-terminal domain is disordered. Thirty-three N-terminal residues are disordered for both domains.

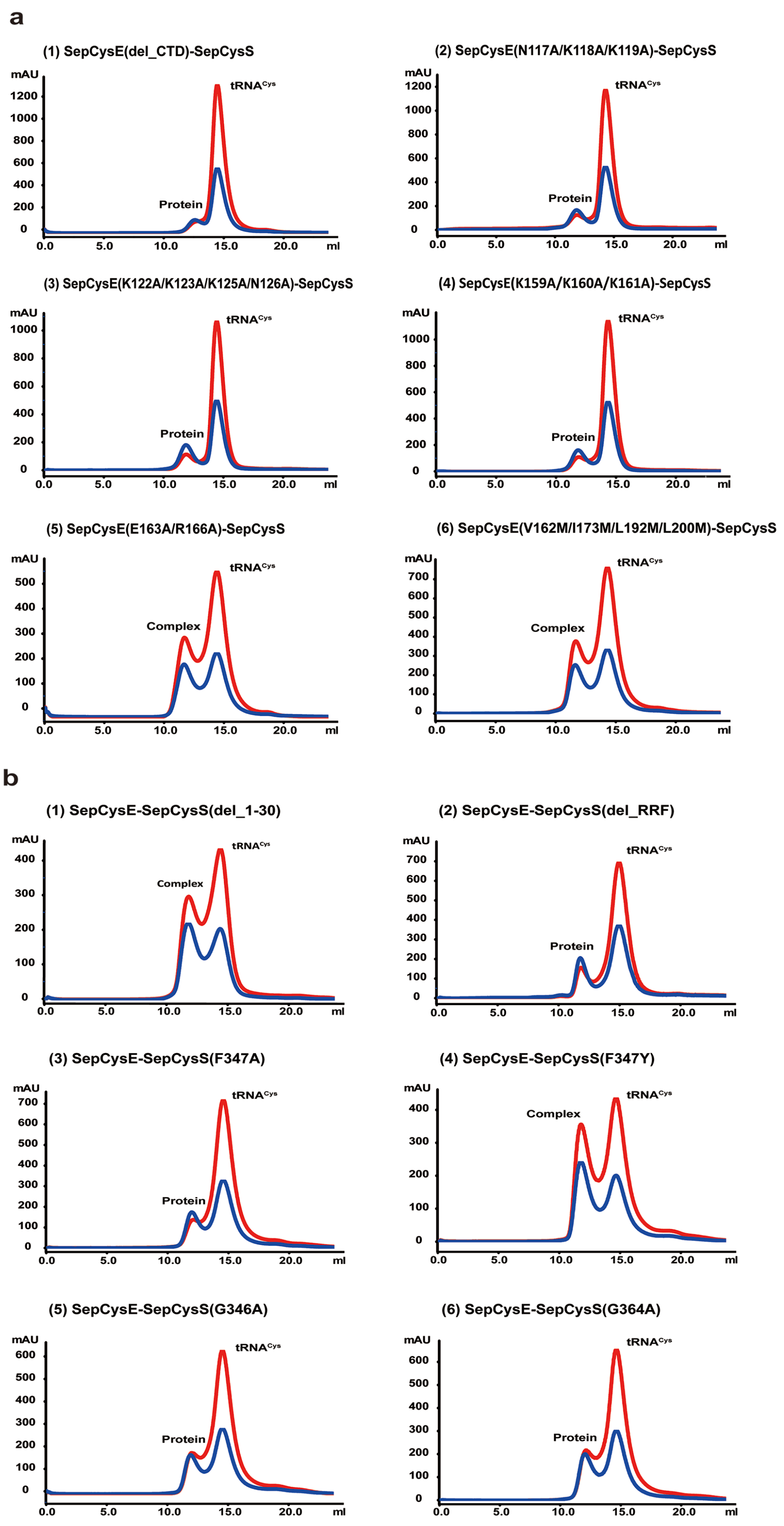

Supplementary Figure 2 Binding experiments of SepCysE-SepCysS mutants to tRNA^{Cys} by gel filtration. Blue and red lines show the absorption at wavelengths of 280 and 260 nm, respectively. **(a)** SepCysE mutants and **(b)** SepCysS mutants.

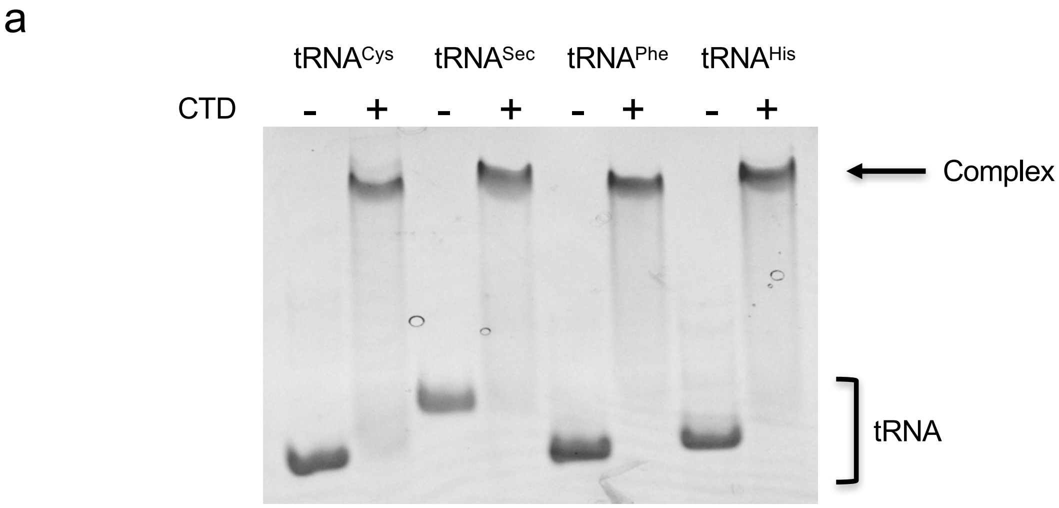

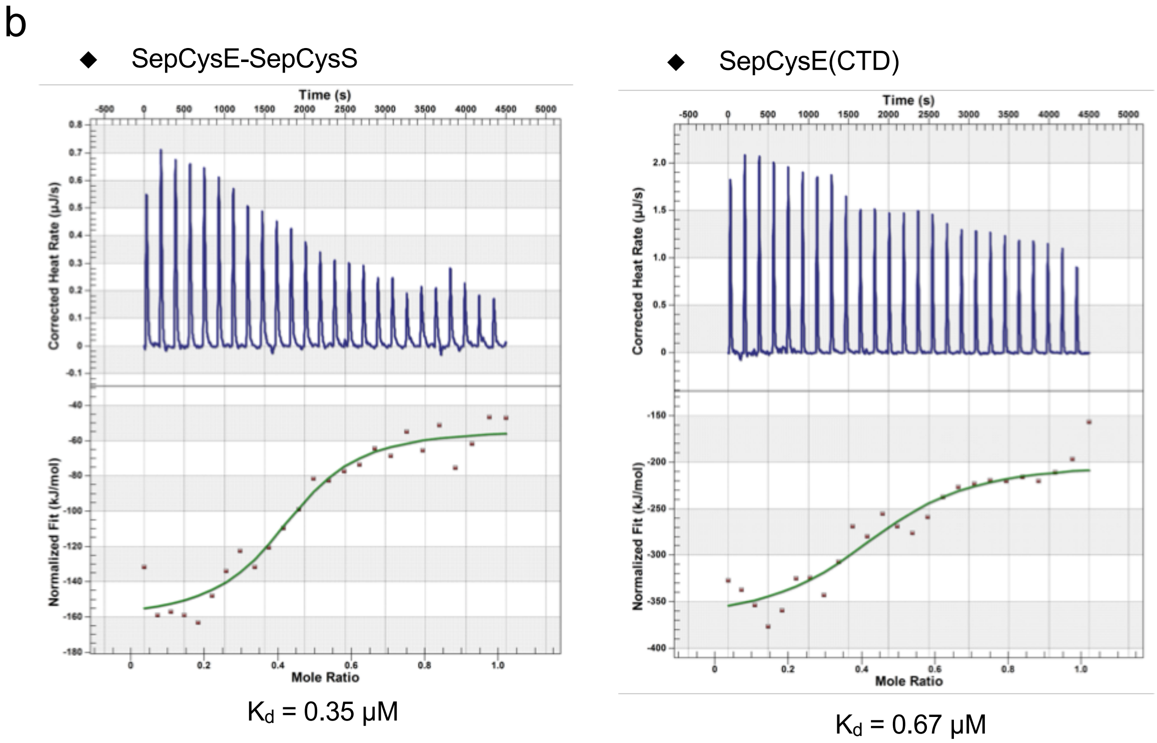

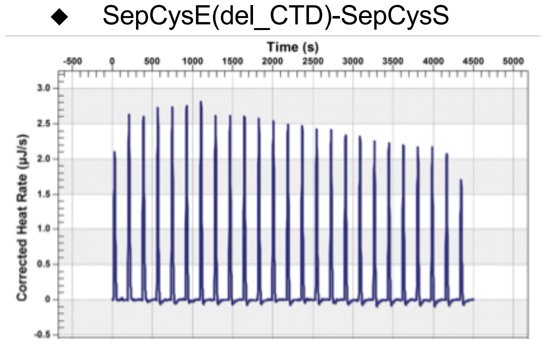

Supplementary Figure 3 SepCysE(CTD) is a non-specific tRNA binding domain. **(a)** Binding experiments of SepCysE(CTD) to various tRNAs by EMSA, showing that SepCysE(CTD) has no specificity to tRNAs. **(b)** Analysis of binding affinity by isothermal titration calorimetry (ITC). In the SepCysE-SepCysS complex, SepCysE(CTD) plays a major role in $tRNA^{Cys}$ binding, although the full complex (SepCysE-SepCysS: K_d = 0.35 μM) is stronger. In contrast, the K_d of SepCysE(del_CTD)-SepCysS to $tRNA^{Cys}$ could not be determined.

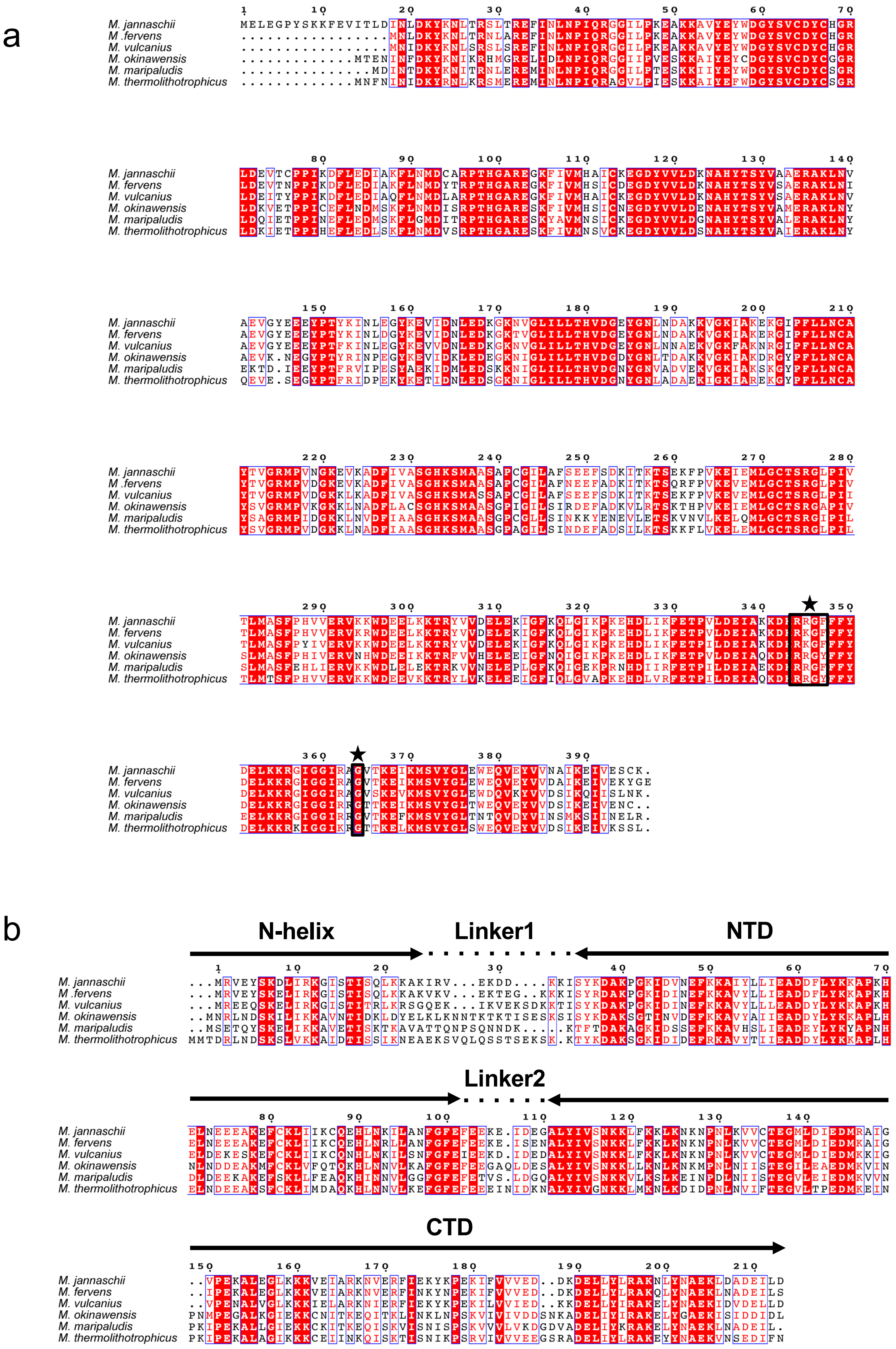

Supplementary Figure 4 Primary sequence alignment of (**a**) SepCysS and (**b**) SepCysE in different species. The residues of SepCysS responsible for U73 recognition of $tRNA^{Cys}$ are black framed and labeled with a star. N-helix, NTD, and CTD of SepCysE from *Methanocaldococcus jannaschii* are marked. The residue numbers corresponding to each domain are as follows: N-helix, 1-24; NTD, 35-101; CTD, 111-213.

a

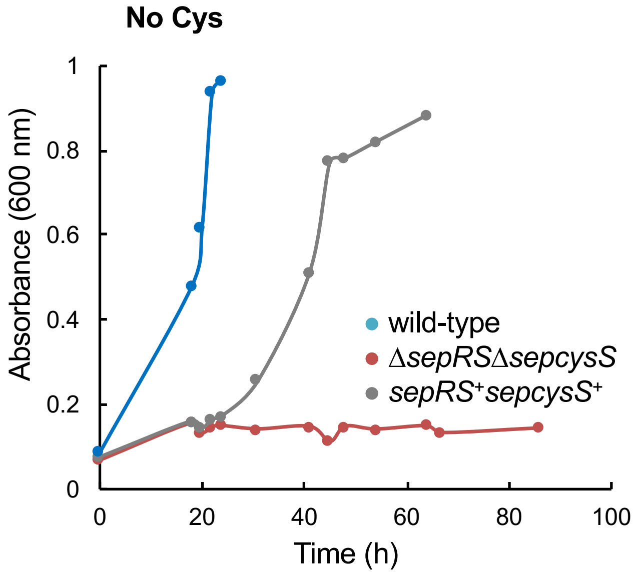

b

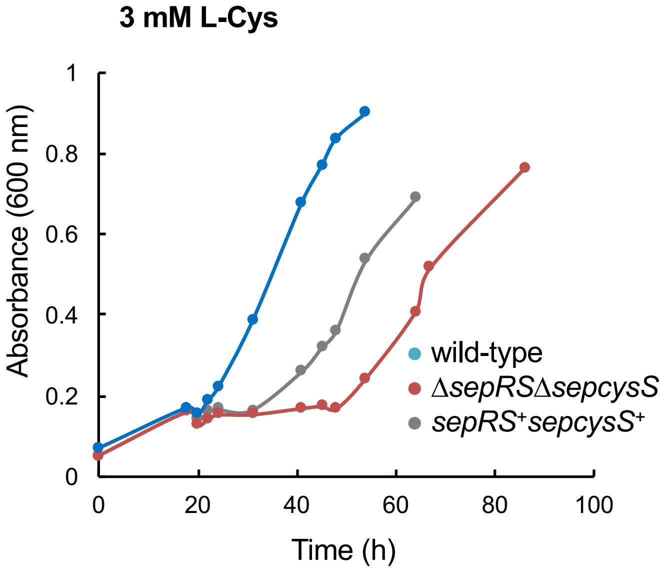

Supplementary Figure 5 Growth of *M. maripaludis* in McFAA medium reduced with 3mM DTT. **(a)** no Cys was added to the medium; **(b)** the medium was supplemented with 3 mM L-Cys. The inocula were 0.8×10^7 cells per 5 ml culture. Each growth curve is a representative of triplicates. These results showed that the addition of cysteine recovered growth of the ΔsepRS/ΔsepcysS mutant strain with both *sepRS* and *sepcysS* deleted, and this double deletion mutant can be complemented with SepRS and SepCysS expressed from a vector.

a

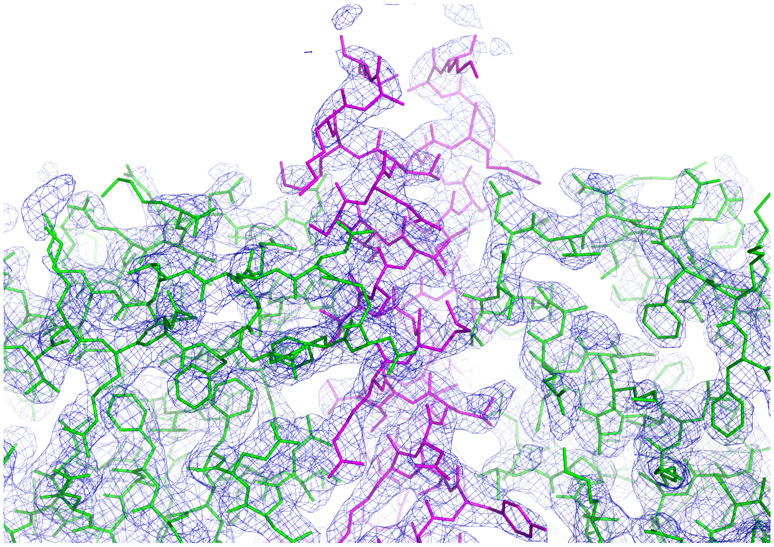

b

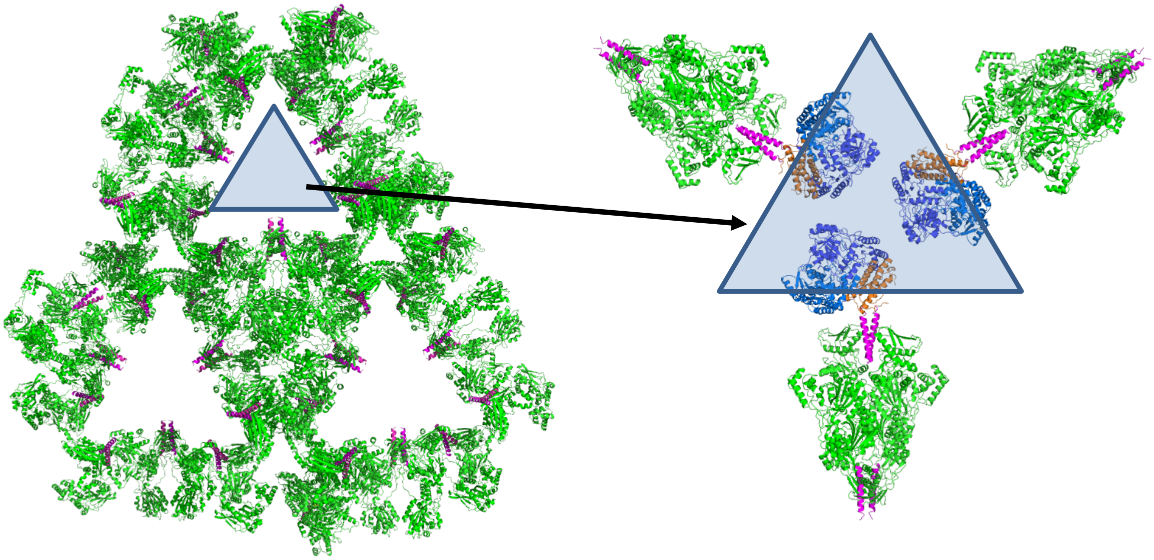

Supplementary Figure 6 Structure of SepRS-SepCysE(N-helix). (a) The contact region of SepRS and SepCysE(N-helix) in transsulfursome structure with $2F_O$-F_C density map contoured by 1.0σ. (b) Crystal packing of transsulfursome (left). The blue triangle shows the empty space large enough to accommodate the structure of disordered SepCysS-SepCysE(right). SepRS and SepCysE(N-helix) are colored by green and pink, respectivety. SepCysS and SepCysE are colored same as in Fig.1.

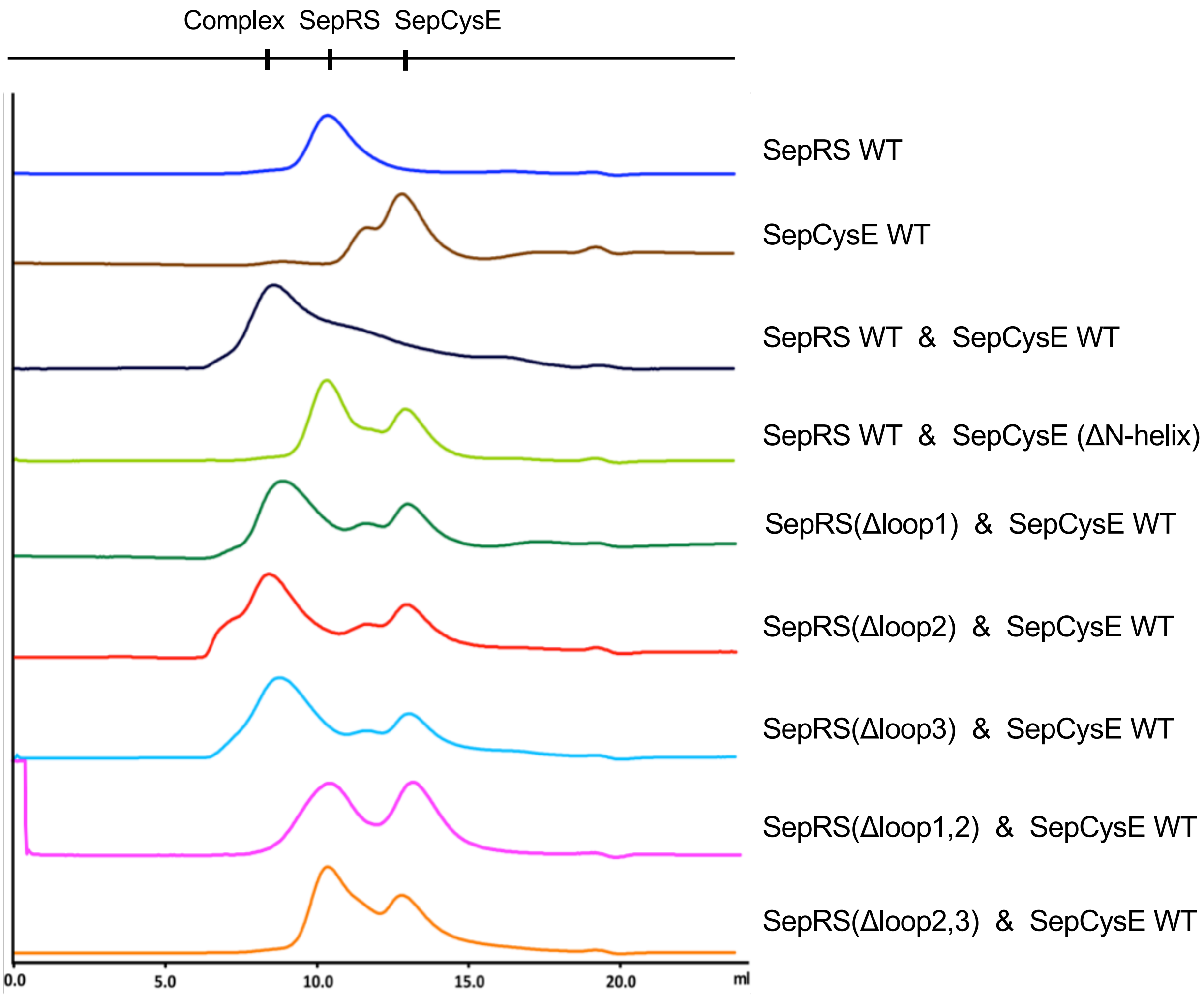

Supplementary Figure 7 Gel filtration experiments for SepRS mutants with SepCysE mutants.

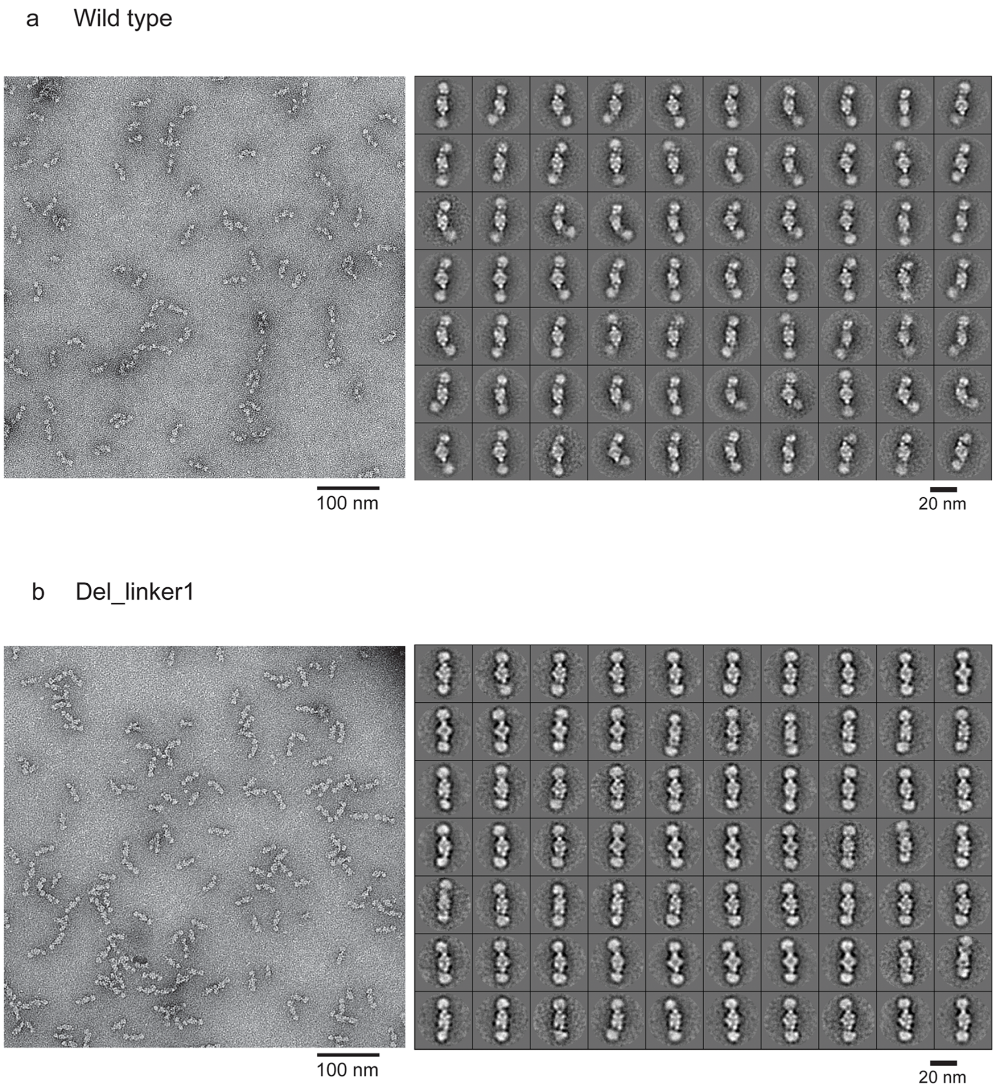

Supplementary Figure 8 Typical micrograph areas (left) and representative class averages (right) of **(a)** wild-type transsulfursome and **(b)** transsulfursome(del_linker1), respectively.

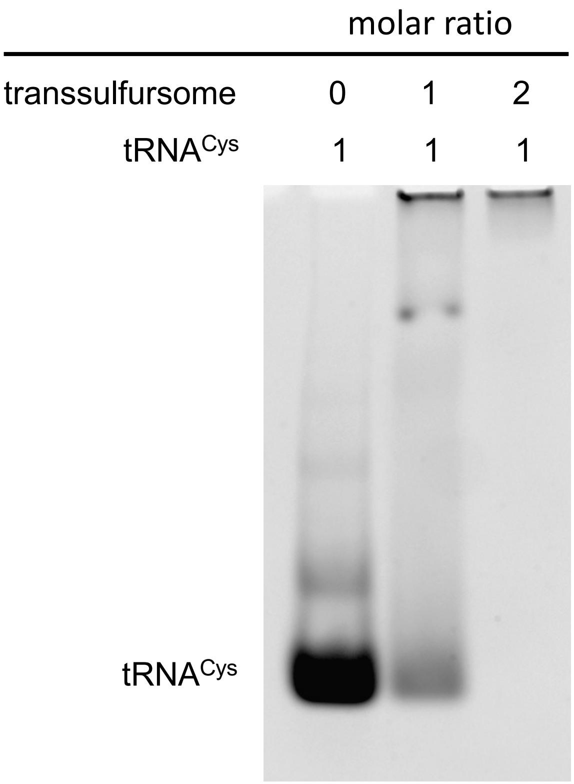

Supplementary Figure 9 Binding experiment of transsulfursome and $tRNA^{Cys}$ by electrophoretic mobility shift assay (EMSA). Transsulfursome and $tRNA^{Cys}$ were incubated with a stepwise increase of transsulfursome ratio. The results show that only half of the $tRNA^{Cys}$ binding sites are used.

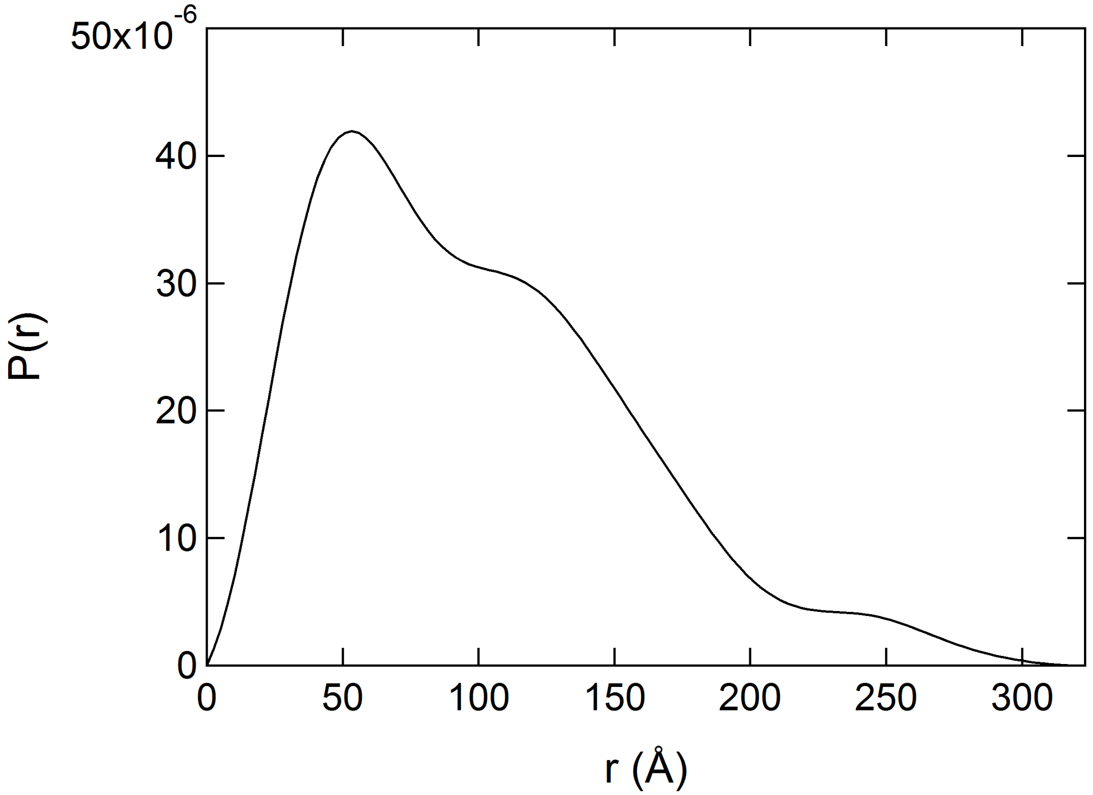

Supplementary Figure 10 The distribution functions P(r) for transsulfursome. The plot shows three peaks reflecting the multidomain of transsulfursome, and a longer tail at large r used to estimate the maximum length (Dmax) of transsulfursome. All analysis results using DATGNOM are given in Supplementary Table 3.

Supplementary Table 1 Ability of SepCysE or SepCysS mutants to support the growth of *M. maripaludis* in the presence of 3 mM L-Cys.

Strains	Proteins expressed from vector	Transformation efficiency[a] (cfu/μg DNA)
Δ*sepRS*Δ*sepcysE*	SepRS + SepCysE(WT)	$(1.5 \pm 0.2) \times 10^5$
Δ*sepRS*Δ*sepcysE*	SepRS + SepCysE(del_linker1)	$(1.1 \pm 0.1) \times 10^5$
Δ*sepRS*Δ*sepcysE*	SepRS + SepCysE(del_linker2)	$(0.9 \pm 0.1) \times 10^5$
Δ*sepRS*Δ*sepcysS*	SepRS + SepCysS (WT)	$(0.7 \pm 0.2) \times 10^5$
Δ*sepRS*Δ*sepcysS*	SepRS + SepCysS(del_RRF)	0

[a]Data are mean ± SDs from four transformations.

Supplementary Table 2 Primer sequences for mutagenesis

Protein	Mutation	Primers (Forward primer; Reverse primer)
M.jannashii SepRS	del_loop1	5'- TACAACTTTGCAAATGAGCTTATTG -3'; 5'- AGGAACTTTATCAACTCTTATCATC -3'
	del_loop2	5'- AGAAGAGTAATTAAAGTAGAAATATTTGAG -3'; 5'- ATTGAATTCCCTTTTAACTTCAAC -3'
	del_loop3	5'- GTGGATGAATTTAAGTTCAGAG -3'; 5'- CTCTTCAATCTTAGCTACTAATTTATAG -3'
M.jannashii SepCysS	del_Nloop	5’- ATGGCTGCCGCGCGGCACCA -3’; 5’- ACAAGGGAATTTATTAACTTAAAT -3’
	del_RRF	5’- GGGTTCTTCTATGATGAGTTGAAG -3’; 5’- CTTATCCTTTTTAGCTATCTCATCC -3’
	G346A	5’- AGGAGAGCGTTTTTCTTCTATGATG -3’; 5’- CTTATCCTTTTTAGCTATCTCATCC -3’
	F347A	5’- AGGAGAGGGGCTTTCTTCTATGATG -3’; 5’- CTTATCCTTTTTAGCTATCTCATCC -3’
	F347Y	5’- AGGAGAGGGTATTTCTTCTATGAT -3’; 5’- CTTATCCTTTTTAGCTATCTCATCC -3’
	G364A	5’- GCTGTTACTAAGGAGATTAAGATG -3’; 5’- TGCTCTAATCCCTCCAATTC -3’
M.maripaludis SepCysS	del_RRF	5’-GGATTCTTCTACGAAGAATTGAAG-3’; 5’-TTTATCTTTTTCTGCAATTTCATC-3’
M.jannashii SepCysE	N117A/K118A/K119A	5’- CAGCTGAGACAATGTATAATGCAC -3’; 5’- CAGCTTTATTTAAAAAGCTTAAAAAC -3’
	K122A/K123A/K125A/N126A	5’- CTTGCAGCCAAAAATCCTAATTTAAAAGTTGTAT -3’; 5’- GGCTGCAAATAACTTTTTATTTGAGACAATGT -3’

	K159A/K160A/ K161A	5’- CAGCGGTTGAGATAGCACGAAAGAAT -3’; 5’- CCGCTAATCCTTCTAATGCTTTTTCTG -3’
	N168A/R171A	5’- GTTGAGGCATTTATAGAAAAATACAAACC -3’; 5’- TGCCTTTCGTGCTATCTCAAC -3’
	del_Nhelix	5'- AGAGTAGAAAAGGATGATAAAAAAATATC -3'; 5'- ATGATTCATGGTATATCTCCT -3'
	del_linker1	5'- TATTTTAGCTTTTTTTAGTTGG -3'; 5'- TACAAAGATGCAAAGCCAGG -3'
M.maripaludis SepCysE	del_linker1	5’- AAGAAGACATTTTCTGATGCAAAATCTGGA AAAATTGATACAAT -3’; 5’- AGTAGCAGAAACTGTTTTTGCCTTTGAAAT CGTTTCTAC -3’
	del_linker2	5’-GCTTTGTATATTGTAAGCAATAAAAAAGT -3’; 5’- TTCGATATCAAATCCAAATCCG -3’

Supplementary Table 3 Details of the result of SEC-SAXS experiment and analysis

Data-collection parameters	
Beamline	PF BL-10C
Beam geometry Beam size	Bent cylindrical mirror + Two slits
	V0.35×H0.55mm
Wavelength (Å)	1.500
Camera distance (mm)	2010
q range ($Å^{-1}$)	0.005 – 0.250
Exposure time (sec)	20
Temperature (K)	293
Structural parameters	
I(0) (cm^{-1}) [from P(r)]	0.076 ± 0.0013
R_g (Å) [from P(r)]	88.7 ± 1.8
I(0) (cm^{-1}) [from Guinier]	0.076
R_g (Å) [from Guinier]	88.3
R_g (Å) calculated from straight model (Fig. 4a)	85
R_g (Å) calculated from bent model (Fig. 4b)	83
D_{max} (Å) [from P(r)]	330.2
Porod volume estimate ($Å^3$)	751,000
Molecular-mass determination	
Estimated MW [from Porod vol.] (kDa)	469
Calculated MW from sequence (kDa)	489
Software employed	
Primary data reduction	SAngler
Data processing	SAngler, DATGNOM
Computation of model intensities	CRYSOL
