# Supplementary material for: Structural basis for tRNA-dependent cysteine biosynthesis
Source: Nat Commun. 2017 Nov 15;8:1521. doi: 10.1038/s41467-017-01543-y (PMC5688128; doi:10.1038/s41467-017-01543-y)
Supplement: Supplementary file 2 — Description of Additional Supplementary Files [file 41467_2017_1543_MOESM2_ESM.pdf]

Description of Supplementary Files

File name: Supplementary Movie 1
Description: Superposition of negative stain EM images shows the dynamic structure of wild type transsulfursome

File name: Supplementary Movie 2
Description: Superposition of negative stain EM images shows reduced flexibility of transsulfursome with deletion of linker 1
