# Supplementary figures and images for: Structural basis for tRNA-dependent cysteine biosynthesis
Source: Nat Commun. 2017 Nov 15;8:1521. doi: 10.1038/s41467-017-01543-y (PMC5688128; doi:10.1038/s41467-017-01543-y)

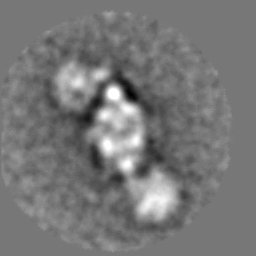

Supplement: Supplementary file 3 — Supplementary Movie 1 [file 41467_2017_1543_MOESM3_ESM.gif]

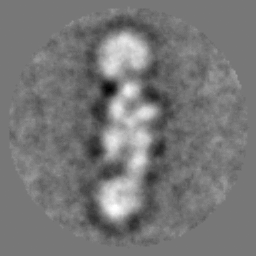

Supplement: Supplementary file 4 — Supplementary Movie 2 [file 41467_2017_1543_MOESM4_ESM.gif]
